# The Roles of Space and Food‐Web Complexity in Mediating Ecological Recovery

**DOI:** 10.1111/ele.70254

**Published:** 2025-11-17

**Authors:** Klementyna A. Gawecka, Matthew A. Barbour, James M. Bullock, Jordi Bascompte

**Affiliations:** ^1^ Department of Evolutionary Biology and Environmental Studies University of Zurich Zürich Switzerland; ^2^ UK Centre for Ecology & Hydrology Penicuik UK; ^3^ Département de Biologie Université de Sherbrooke Sherbrooke Québec Canada; ^4^ UK Centre for Ecology & Hydrology Wallingford UK

**Keywords:** ecological restoration, food webs, landscape dynamics, metacommunity dynamics, species recovery, trophic interactions

## Abstract

Landscape‐scale ecological restoration is a key strategy for halting and reversing biodiversity decline. However, ensuring the long‐term sustainability of restoration efforts requires guiding the recovery of complex ecological systems with many interdependent species at a landscape scale. Due to these challenges, our understanding of recovery trajectories remains limited. Using metacommunity models and experiments, we explore how the spatial configuration of communities and food‐web complexity jointly influence species recovery at different spatial scales. We find that the number and spatial placement of communities affect the colonisation of empty habitat patches, but do not influence population recovery in patches where communities are introduced. Food‐web complexity reduces the recovery of lower trophic levels. However, this negative effect may be partially mitigated at higher levels of food‐web complexity. Our results demonstrate that the joint consideration of spatial configuration and species interactions could enhance the effectiveness of restoration actions.

## Graphical abstract

We investigate how spatial configuration and food‐web complexity influence species recovery during landscape‐scale ecological restoration. We find that while the spatial arrangement of communities affects colonisation of empty patches, it does not impact recovery in already populated patches. Food‐web complexity can slow recovery of lower trophic levels, though this effect may lessen with increasing complexity.

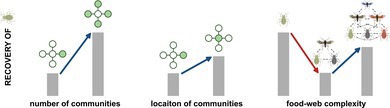

## 1 Introduction

To ‘bend the curve’ of biodiversity loss, we must restore our degraded or lost natural habitats (Leclère et al. 2020). To ensure the persistence of species, restoration activities need to focus on landscapes—that is, metapopulations and metacommunities—rather than on individual sites (Isaac et al. 2018; Bullock et al. 2002). In particular, to promote functioning ecosystems, we must restore ecological communities of multiple interacting species (Oliver et al. 2015; Tylianakis et al. 2010). Together, spatial extent and community complexity can promote long‐term sustainability and resilience (Bullock et al. 2022).

Yet, as systems become more extensive and complex, predicting their dynamics becomes increasingly difficult. Ecological recovery is often assessed at single locations and at a limited number of timepoints by measuring species abundance or richness (e.g., Escobar et al. 2025; Hordijk et al. 2024; Banin et al. 2023; Resch et al. 2022). Success is then determined by comparing these measures to a chosen reference state (Atkinson et al. 2022). However, complex systems can be highly dynamic and follow nonlinear trajectories (Sutheimer et al. 2025; Aoyama et al. 2022). Importantly, these trajectories can vary across space, depending on the local biotic (e.g., species abundances) and abiotic (e.g., habitat type or location within the landscape) conditions. Understanding these spatially structured recovery trajectories and their drivers is vital for guiding restoration actions (Montoya 2021; Suding 2011), and hence for effective restoration planning.

One key factor shaping recovery is spatial configuration. The arrangement and connectivity of habitat patches govern species dispersal and colonisation processes. Thus, the spatial structure of the landscape influences local community dynamics (Bowler and Benton 2005, 2009), species spread across the landscape (Rayfield et al. 2023; Gawecka and Bascompte 2023; Saade et al. 2022; Gilarranz et al. 2017) and metacommunity structure (Bertellotti et al. 2023), stability (Arancibia 2024) and persistence (Bhandary et al. 2025; Li et al. 2023; Arancibia and Morin 2022; Gilarranz and Bascompte 2012). Despite its clear significance, a critical question remains: how does spatial configuration affect recovery trajectories?

Restoration practice often focuses on promoting a target species or enhancing species diversity (Pettorelli and Bullock 2023; Brudvig 2011). However, species in a community are interdependent, through direct or indirect interactions. Species interactions influence community and metacommunity dynamics (Bastolla et al. 2009; May and Hassell 1981), stability (Firkowski et al. 2022; Rohr et al. 2014; Thébault and Fontaine 2010) and persistence (Domínguez‐Garcia et al. 2024; Gaiarsa and Bascompte 2022). The recovery of one species can have profound impacts on others (Gawecka and Bascompte 2021; Horn et al. 2020; Baker et al. 2019) and this effect depends on the community and landscape context (Twining et al. 2022). Yet, species interactions are rarely considered in restoration practice (Hallett et al. 2023). If restoration's goal is to rebuild ecological complexity, we must understand how species interactions affect recovery trajectories across landscapes.

**FIGURE 1:**
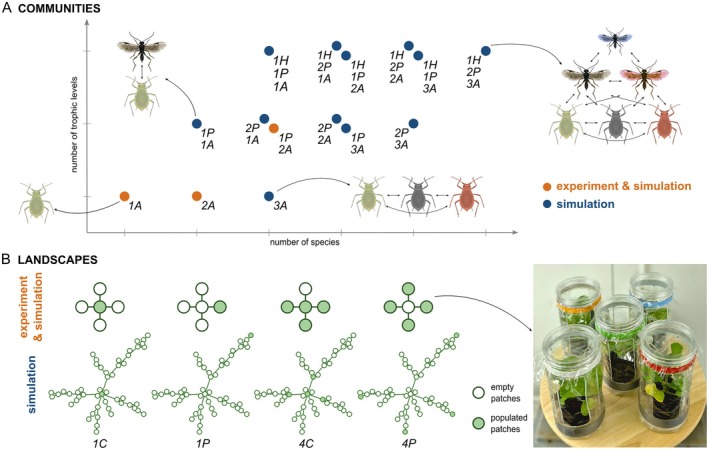
Our model system. (A) 15 communities increasing in food‐web complexity from a single aphid species to three aphid, two parasitoid wasp and one hyperparasitoid wasp species. The experimental communities are shown in orange. The communities are labelled according to the number of aphid (*A*), parasitoid (*P*) and hyperparasitoid (*H*) species. The aphid icons correspond to *Brevicoryne brassicae* (green), *Lipaphis erysimi* (grey) and *Myzus persicae* (red). The parasitoid wasp icons represent *Diaeretiella rapae* (grey) and *Aphidius colemani* (red). The hyperparasitoid wasp is *Alloxysta fuscicornis*. (B) Experimental landscapes consist of five habitat patches connected in a star configuration. Larger simulated landscapes consist of 50 habitat patches connected to form a scale‐free network. Initially, only selected patches contain a plant and an insect community (*populated* patches, green filled circles). Other patches initially contain only a plant (*empty* patches, white filled circles). Aphid icons adapted from ‘Green peach aphid’ (DBCLS, via Wikimedia Commons, CC BY 4.0, colours modified). (Hyper)parasitoid icons adapted from ‘ *Chaoa flavipes* ’ (Fernandez‐Triana et al. 2020, via Wikimedia Commons, CC0 1.0, colours modified). Photo by K. A. Gawecka.

Here, we study the landscape‐scale recovery of species embedded in communities. Specifically, we investigate how recovery trajectories are affected by (1) spatial configuration—the number and location of introduced communities and (2) food‐web complexity, defined by the number of species and trophic levels (Bullock et al. 2022). We assess recovery at both local (habitat patch) and regional (landscape) scales. As examining such processes robustly and understanding their underlying mechanisms is challenging in field settings, we combine modelling with controlled experiments (e.g., Gilarranz et al. 2017). First, we develop a parameterised metacommunity model and test its predictions through an experiment using three insect communities differing in food‐web complexity, within a five‐patch landscape. Second, we use the model to generalise the findings and extrapolate them to more complex landscapes and communities (Figure 1).

## 2 Methods

### 2.1 Model System

Host‐parasitoid communities are a great model system for studying real‐world multitrophic food webs, both from theoretical and experimental perspectives (May and Hassell 1981). These systems are ecologically important as they play key roles in natural and agricultural ecosystems, where parasitoids serve as biological control agents. We selected a naturally occurring food web comprising a plant (radish, *Raphanus sativus* ), three aphid species (cabbage aphid *Brevicoryne brassicae* , turnip aphid *Lipaphis erysimi* and green peach aphids *Myzus persicae* ), two parasitoid wasp species (*Diaeretiella rapae* and *Aphidius colemani*) and a hyperparasitoid wasp (*Alloxysta fuscicornis*) (Figure 1A).

The aphid species differ in their competitive abilities, with *B. brassicae* being the weakest competitor and *M. persicae* the strongest (Barbour et al. 2022). The parasitoid wasp species differ in their host preferences. *D. rapae* has the highest attack rate on *B. brassicae* and the lowest on *M. persicae* (Barbour et al. 2022). *A. colemani* has a strong preference for *M. persicae* , although it has been observed to parasitise *B. brassicae* and *L. erysimi* as well (Finke and Snyder 2008). The hyperparasitoid wasp *A. fuscicornis* attacks the larvae of *D. rapae* at a higher rate than *A. colemani* (Barbour, unpublished data). Here, we focus on the response of aphid *B. brassicae* due to its poor competitive abilities and high parasitisation rate by *D. rapae* suggesting its recovery may be more uncertain.

This system enables us to construct food webs that range in complexity, both in terms of the number of species and trophic levels (Figure 1A). In the experiment, we used three communities: a single aphid species (community *1A* with *B. brassicae* only), two aphid species (community *2A* with *B. brassicae* and *L. erysimi* ) and two aphid and one parasitoid species (community *2A‐1P* with *B. brassicae* , *L. erysimi* and *D. rapae*). For further metacommunity model simulations, we created an additional 12 food webs with up to six species and three trophic levels (Figure S7).

We considered community dynamics in fragmented landscapes composed of discrete habitat patches, and with connections between certain patches representing possible dispersal routes (Figure 1B). Our experimental landscape comprised five habitat patches in a star configuration with one central patch and four peripheral patches. A habitat patch was represented by a cylindrical, transparent polyethylene container (diameter 10 cm, height 20 cm). Four side openings and one in the lid were covered with transparent cellophane for air exchange, while drainage holes in the bottom were covered with nylon mesh to prevent insect escape. The containers housed a 7 cm × 7 cm × 6 cm plant pot. The patches were connected with a silicone tube (diameter 1 cm) at the top of the container. A thread running through the tube further enabled the insects to migrate between the patches. This setup is particularly suited for modelling metacommunities of aphids—the focal species of this study—as their limited mobility means that movement between patches likely reflects true dispersal. By contrast, (hyper)parasitoids are more mobile, and their movement between patches is more likely to represent foraging. Such differences in movement scales among species are also observed in natural landscapes (e.g., Van Nouhuys and Hanski 2002).

Additionally, we simulated larger landscapes consisting of 50 habitat patches. We connected the patches such that they formed a scale‐free network (where the number of connections per patch follows a power law distribution). This resulted in a few highly connected patches (hereafter central) and many poorly connected ones (hereafter peripheral). Such landscape structures have been found in natural systems (e.g., Prima et al. 2018; Minor and Urban 2008).

In both the experiment and the model simulations, we considered four different spatial configurations of initial placement of communities: one central patch (landscape *1C*), one peripheral patch (landscape *1P*), four central patches (landscape *4C*) and four peripheral patches (landscape *4P*) (Figure 1B). We refer to the patches initially containing a community as *populated*, and patches with initially only a plant as *empty*. In the experiment, all patches contained a single, two‐week‐old plant representing the patch's ‘habitat’.

We applied these community and landscape treatments in a fully factorial design, resulting in 12 experimental combinations (three communities and four landscapes) and 60 simulated combinations (15 communities and four landscapes). We replicated each combination five times in the experiment and 100 times in the simulations.

### 2.2 Metacommunity Model

We developed a spatially explicit model based on the mass‐effect metacommunity paradigm (Leibold et al. 2004). It describes the local dynamics of our communities and dispersal across the landscape. Its general form resembles other discrete‐time mass‐effect models with Lotka‐Volterra‐type competition and predation (e.g., Thompson and Gonzalez 2017). However, we chose the specific functions such that the model reproduced the observed dynamics of our experimental communities (see metacommunity model description and parameterisation in Supporting Information, Figures S1–S6). This approach balances the model's generality and precision (Levins 1966). The population size of aphid species i in patch k at time t is given by:

(1) $$A_{i,k}^{t+1}=A_{i,k}^{t}\exp\left(r_{i}-\sum_{j=1}^{n}\alpha_{\mathrm{ij}}A_{j,k}^{t}\right)-M_{i,k}^{t}-E_{i,k}^{t}+I_{i,k}^{t}$$

The first term describes density‐dependent intrinsic growth, intraspecific competition and interspecific competition with aphid species j. $r_{i}$ is the intrinsic growth rate, $\alpha_{\mathrm{ij}}$ is the intraspecific (when j=i) or interspecific competition coefficient (when j≠i), and n is the number of aphid species. We found that this exponential form of the logistic growth model (e.g., Agrawal 2004) best reproduced the experimental data (Figures S1, S3, S6). The second term represents the mortality due to parasitism and depends on the parasitoid density. Based on experimental observations, we adopted a piecewise linear saturating function which mimics a Type II functional response (Equation S3, Figure S4). The final two terms describe density‐dependent emigration from patch k (Equation S4, Figure S2) and immigration into patch k from adjacent patches (Equation S5), respectively.

The parasitoid and hyperparasitoid population sizes were modelled as a balance between births, deaths, emigration and immigration (Equations S6–S8). Parasitoid births depend on the density of the hyperparasitoid which lays eggs inside the parasitoid's larvae, thus reducing the number of emerged parasitoids. We adopted the same form of functional response for parasitoid‐hyperparasitoid interaction as for aphid‐parasitoid. We provide the details of all model functions in the SI.

We parameterised the model using a series of experiments on *B. brassicae* , *L. erysimi* and *D. rapae*, each designed to enable the determination of a parameter(s) (Table S1). For example, to estimate the intrinsic growth rate and intraspecific competition of aphid *B. brassicae* , we (1) experimentally measured its population size in a single patch over time, (2) fitted a linear model to per capita growth rate (in terms of log‐transformed differences) versus its population size and (3) obtained confidence intervals of the intercept (intrinsic growth rate) and slope (intraspecific competition). For the other species in our simulated food webs ( *M. persicae* , *A. colemani* and *A. fuscicornis* ), we estimated model parameters based on the values determined for *B. brassicae* , *L. erysimi* or *D. rapae*, previous studies and observations on this experimental system (Barbour et al. 2022), and expert opinion. We provide more details on the parameterisation procedure and model parameter values in the SI.

We used the metacommunity model to perform two sets of simulations. First, we simulated the dynamics of the three experimental communities on the 5‐patch landscapes. Second, to check the validity of the findings in more complex systems, we simulated the dynamics of all 15 communities on 50‐patch landscapes (Figure 1). At the start of the simulation, we placed one of the communities, in patches shown as *populated* in Figure 1B. The initial population sizes were ten for each aphid species and one female for each parasitoid and hyperparasitoid species, where applicable. We simulated the dynamics for 26 days for comparability with the experiment (see below), tracking population sizes of all species in all patches through time.

### 2.3 Experimental Procedure

The plants were seeded two weeks prior to the start of the experiment and grown in a greenhouse. We reared aphids and parasitoid wasps in mesh cages in a climate chamber set to 22°C, 50% humidity and 16 h photoperiod. Aphids sourced for the experiment were maintained on the same radish species as used in the experiment. The parasitoid wasps were reared on a non‐experimental aphid species ( *M. persicae* ). For more details on the insect colonies, refer to Barbour et al. (2022).

At the start of the experiment, we placed ten aphids of each species on plants inside the relevant containers (i.e., *populated* patches, Figure 1B). In the case of community *2A‐1P*, we transferred a single one‐day‐old, mated female parasitoid wasp into the same containers as the aphids. We placed the experimental units on trays in the climate chamber (set to 22°C, 50% humidity and 16 h photoperiod) for the duration of the experiment. We positioned the units in random orientations and locations within the climate chamber and shuffled them every week.

We counted aphids of each species in each container twice a week (every 3 or 4 days, see Figures S8, S9 for aphid count time series) and watered the experimental units once per week. The experiment ran for 26 days, which covered approximately four generations of aphids, two generations of parasitoid wasps and the lifespan of the plants.

### 2.4 Recovery Measure

To quantify the recovery trajectory, we computed the recovery credit (Marjakangas et al. 2018; Hanski 2000) which is analogous to the recovery debt (Moreno‐Mateos et al. 2017), but represents the surplus in population or metapopulation size. We defined our recovery credit as the area under the curve of the population or metapopulation size against time (Figures S8, S12, S17). In other words, our recovery credit is an integrative measure of (meta)population size through time. We calculated this credit for our focal aphid species, *B. brassicae* , at two spatial scales. At the local scale, we considered the population size in each patch, differentiating between initially *populated* and *empty* patches (Figure 1B). This allowed us to study the local dynamics within introduced populations and colonisation of the rest of the landscape. At the regional scale, we evaluated the recovery of metapopulation size—the sum of population sizes across all patches, which represents the overall species recovery across a landscape.

### 2.5 Statistical Analysis

We performed three‐way ANOVA to assess the effects of (1) the number, (2) the location and (3) the food‐web complexity of the introduced communities on the recovery credit. We carried out separate tests for the recovery credit of (a) populations in *empty* patches, (b) populations in *populated* patches and (c) metapopulations of each aphid species. In the case of local population recovery, we considered the average recovery credit across all initially *empty* or *populated* patches in each landscape.

To ensure normality and include zero values of recovery credit, we applied $\ln\left(x+1\right)$ transformation to the calculated recovery credit (for untransformed results, see Figures S11, S14). We report the results in terms of average effect as a percentage change, and provide the ANOVA tables in SI (Tables S2–S10). We performed all statistical analyses and model simulations in R version 4.4.0 (R Core Team 2020).

## 3 Results

### 3.1 Experimental Landscapes and Communities

We first present the experimental results from three insect communities in five‐patch landscapes (Figure 1) and compare them with predictions from the metacommunity model.

### 3.2 Effect of Spatial Configuration

**FIGURE 2:**
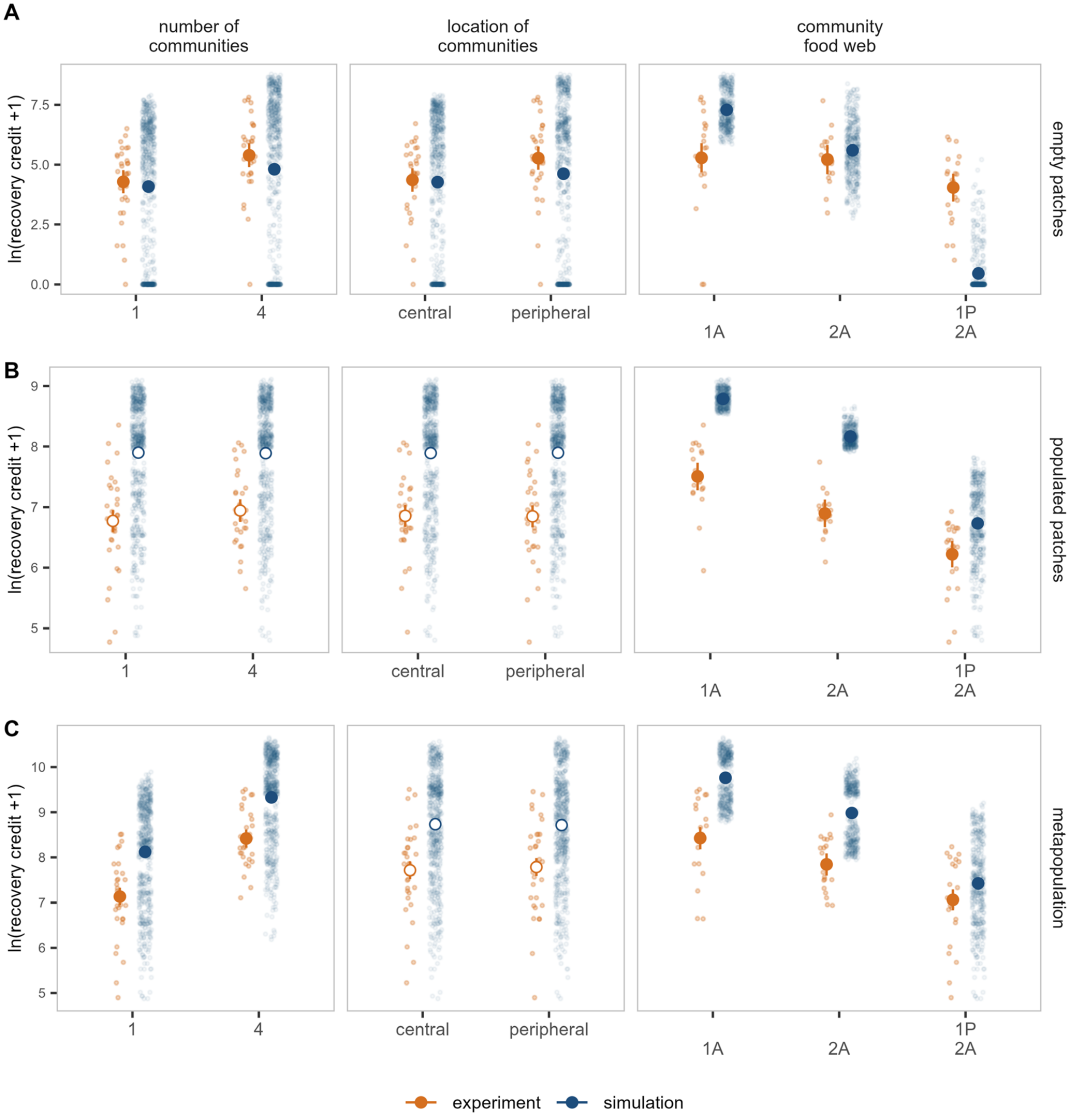
Recovery of our focal aphid (A) in initially *empty* patches, (B) in initially *populated* patches and (C) *metapopulation*. Panels show the effects of the initial number of communities (left), location of initial communities (middle) and community food web (right) on the recovery credit ($\ln\left(x+1\right)$ transformed). Experiment and model simulations are shown in orange and blue, respectively. Smaller points represent recovery credit calculated from model simulations or experimental data. Larger points and vertical lines depict average linear model predictions and their 95% confidence intervals, respectively. Full and empty points indicate statistically significant and nonsignificant effects, respectively.

Increasing the **number of introduced communities** has a positive effect on the recovery of aphid populations in initially *empty* patches and its *metapopulation*, but does not influence population recovery in initially *populated* patches (Figure 2, left panels). Introducing four communities compared to one increases recovery credit in initially *empty* patches by 26% (*F*_1,50_ = 10.8, *p* = 0.0019) and 18% (*F*_1,1188_ = 270, *p* < 0.001) according to our experiment and model simulations, respectively. At the *metapopulation* scale, this increase is 18% (*F*_1,50_ = 87.7, *p* < 0.001) and 15% (*F*_1,1188_ = 2528, *p* < 0.001) based on the experimental and simulation results, respectively.

The **location of introduced communities** affects population recovery in initially *empty* patches, but not the recovery in initially *populated* patches or at the *metapopulation* scale (Figure 2, middle panels). Introducing communities into peripheral rather than central patches increases recovery credit in initially *empty* patches by 21% (*F*_1,50_ = 6.7, *p* = 0.012) and 8% (*F*_1,1188_ = 61, *p* < 0.001) according to the experiment and model simulation, respectively. Furthermore, we find a significant interaction between the number and location of communities (experiment: *F*_1,50_ = 6.2, *p* = 0.016, Figure S11; simulation: *F*_1,1188_ = 269, *p* < 0.001, Figure S14). Post hoc comparisons reveal that the effect of the number of initial communities is substantial only in landscapes where communities are introduced into peripheral patches (landscapes *1P* and *4P*). In turn, community location has a strong effect in landscapes with four introduced communities (landscapes *4C* and *4P*), but a negligible effect in landscapes with one introduced community (landscapes *1C* and *1P*).

In summary, we find that spatial configuration affects the recovery in initially *empty* but not initially populated patches. Our metacommunity model allows us to decompose the contribution of local community processes and dispersal to the change in population size at a given time (Figure S15). First, we find that the initially *populated* patches act as sources, with emigration outweighing immigration. Conversely, the initially *empty* patches are sinks with net immigration. Second, the absolute contribution of dispersal to the change in population size tends to be smaller in the initially *populated* than in *empty* patches. This suggests that the population growth in the initially *populated* patches is driven by local intra‐ and inter‐specific dynamics, rather than immigration and emigration.

### 3.3 Effect of Food‐Web Complexity

We find a significant negative effect of food‐web complexity on recovery across all scales (Figure 2, right panels): initially *empty* patches (experiment: *F*_2,50_ = 5.4, *p* = 0.0072; simulation: *F*_2,1188_ = 8662, *p* < 0.001), initially *populated* patches (experiment: *F*_2,50_ = 32.5, *p* < 0.001; simulation: *F*_2,1188_ = 2821, *p* < 0.001) and *metapopulation* (experiment: *F*_2,50_ = 32.1, *p* < 0.001; simulation: *F*_2,1188_ = 3281, *p* < 0.001). For example, at the *metapopulation* scale, the addition of another aphid species (comparing communities *1A* and *2A*) decreases our focal aphid's recovery credit by 7% in the experiment and 8% in the simulations. The inclusion of a parasitoid (comparing communities *2A* and *2A‐1P*) causes a further reduction in recovery of 10% and 17% according to the experiment and simulations, respectively. In initially *populated* patches, we find very similar effect sizes as at the *metapopulation* scale. In initially *empty* patches, our experiment shows a smaller, although significant, effect of food‐web complexity than the model (Figure 2A, right panel).

To understand why food‐web complexity affects recovery across scales, we consider the temporal trends in the contributions of community processes and dispersal to population change (Figure S16). Food‐web complexity strongly influences the local community dynamics, and thus the recovery in initially *populated* patches. However, since aphids disperse when they reach a certain density, food‐web complexity also affects the time at which they begin to disperse, and thus, the recovery in initially *empty* patches. Once dispersal begins, the spatial configuration starts to play a role. Furthermore, this indirect effect of food‐web complexity together with differences in species dispersal abilities (Table S1) results in spatio‐temporal variation in local food webs in initially *empty* patches (Figures S10, S13). For example, in the most complex experimental community (*2A‐1P*), local food webs consisting of aphid *L. erysimi* are more prevalent than communities with both aphid species.

## 4 Simulated Landscapes and Communities

The close agreement between the model simulations and the experimental results supports using the model for more complex systems involving larger landscapes and communities (Figure 1).

### 4.1 Effect of Spatial Configuration

**FIGURE 3:**
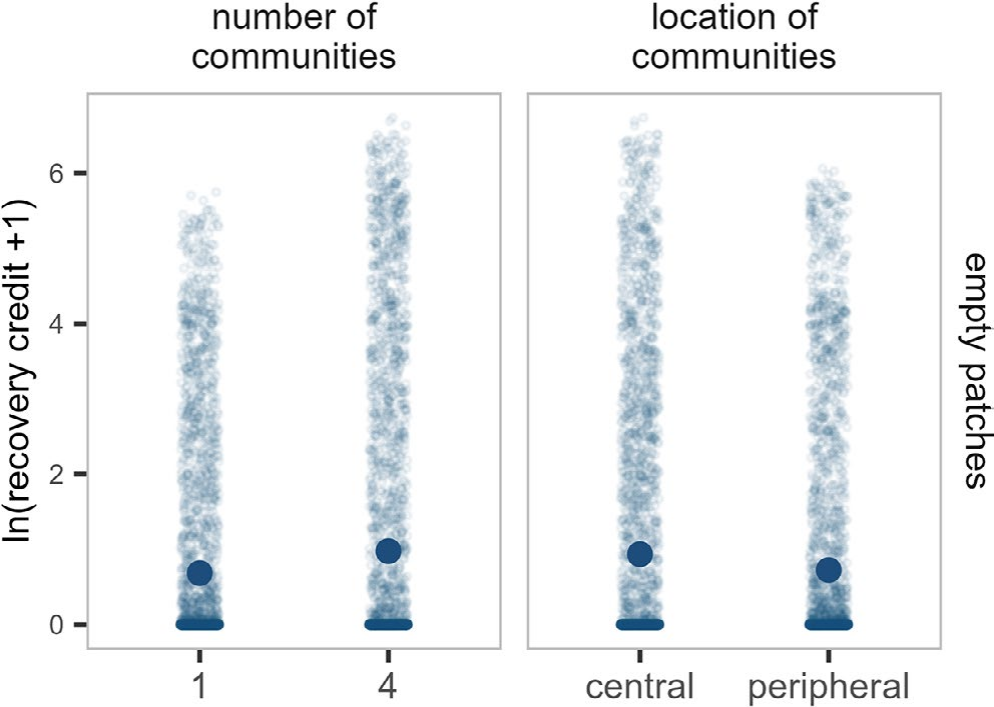
Recovery of our focal aphid in initially *empty* patches in model simulations on larger landscapes and communities. Panels show the effects of the initial number of communities (left) and location of initial communities (right) on the recovery credit ($\ln\left(x+1\right)$ transformed). Smaller points represent recovery credit calculated from model simulations on larger landscapes and 15 insect communities. Larger points depict average linear model predictions, with full points indicating statistically significant effects.

We find a positive effect of increasing the **number of introduced communities** on the recovery in initially *empty* patches and at the *metapopulation* scale, but no effect in initially *populated* patches (Figures 3 and S18). This pattern is consistent with the response observed in the smaller 5‐patch landscape, suggesting that the result is generalizable across different landscape sizes.

The **location of introduced communities** does not significantly affect the recovery in initially *populated* patches or at the *metapopulation* scale (Figure S18). This is also in agreement with the smaller landscapes. However, in contrast to the 5‐patch landscape, the recovery credit in initially *empty* patches is lower when communities are introduced into peripheral compared to central patches (Figure 3). In the 5‐patch landscape, the peripheral patches are directly connected to the central patch, whereas in the 50‐patch landscape, the peripheral and central patches are separated by many patches. As such, colonisation of the initially *empty* patches is substantially easier in the smaller landscape than in the larger one. Thus, the effect of the location of introduced communities depends on the size of the landscape.

### 4.2 Effect of Food‐Web Complexity

**FIGURE 4:**
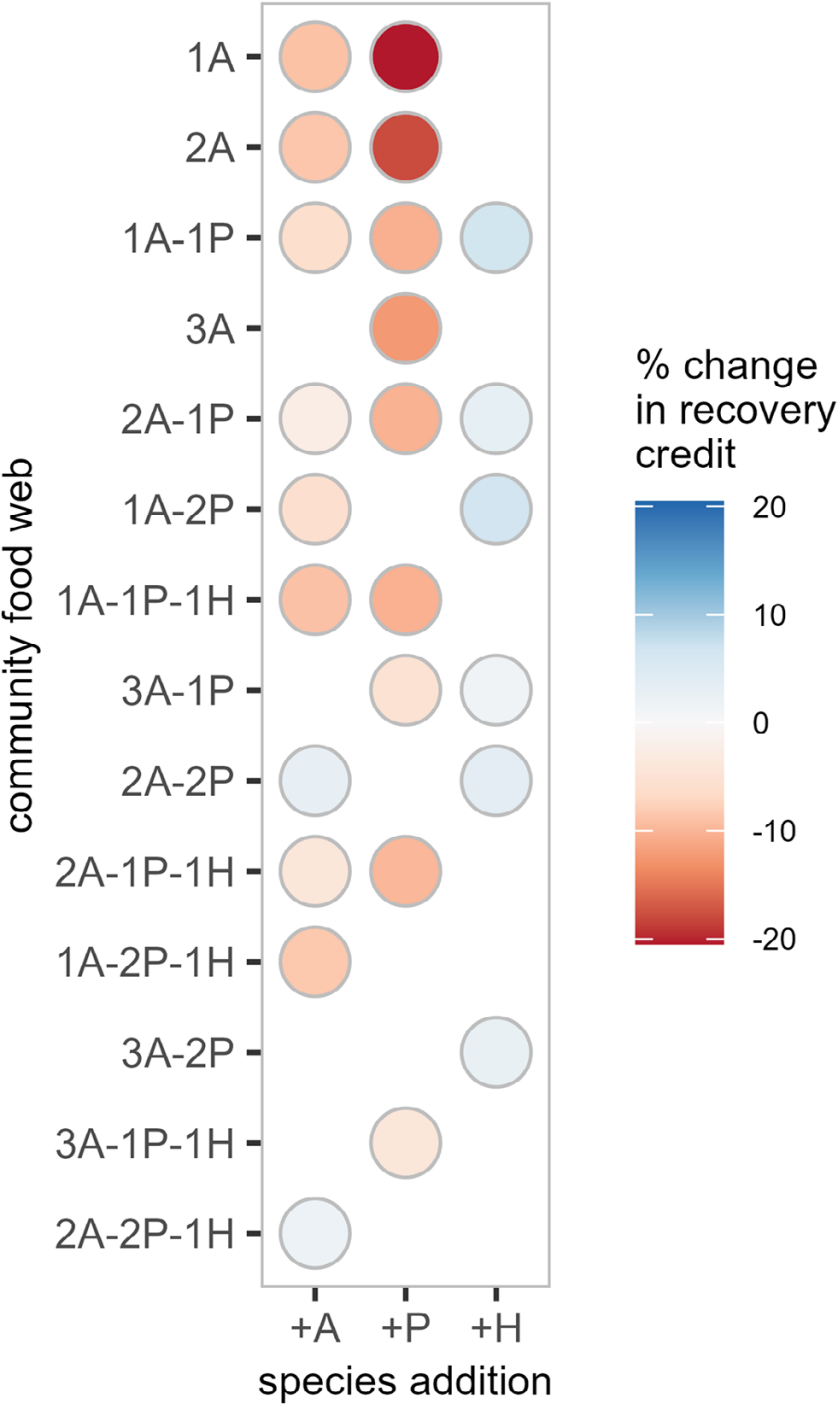
Effect of increasing food‐web complexity on our focal aphid's recovery in model simulations on larger landscapes and communities. The colours indicate the average percentage change in the *metapopulation* recovery credit due to the addition of an aphid (+A), a parasitoid (+P) or a hyperparasitoid (+H) species. For example, for community *1A*, the average percentage change due to the addition of an aphid or a parasitoid is based on the comparison of communities *1A* and *2A* or *1A* and *1A‐1P*, respectively. The average percentage change is obtained from linear model predictions depicted in Figure S18. Communities are ordered by increasing number of species and trophic levels from top to bottom.

Generally, increasing food‐web complexity reduces recovery credit across all scales (Figure 4, Figure S18). However, the addition of certain species has a positive effect on aphid's recovery. Specifically, hyperparasitoid's presence increases the recovery credit in all simulated food webs, by as much as 7% (comparing communities *1A–2P* and *1A–2P–1H*). Moreover, adding a third aphid species can also boost the focal aphid's recovery, despite it being the strongest competitor out of the three aphid species. Yet, we find that this occurs in the more complex communities with two parasitoid species (comparing communities *2A–2P* and *3A–2P*, or *2A–2P–1H* and *3A–2P–1H*).

## 5 Discussion

We find that both spatial configurations—the number and location of introduced communities—and food‐web complexity affect species recovery. Importantly, our model simulation and experimental results largely agree on these patterns, supporting the generality of the findings. Yet, the importance of these factors depends on the process of interest—local population growth or landscape colonisation. Both processes are key to ecological restoration, and our analysis suggests important consequences for nature recovery actions.

### 5.1 The Role of Spatial Configuration

#### 5.1.1 Colonisation of Empty Patches

Increasing the number of introduced communities enhances recovery both in initially *empty* patches and at the *metapopulation* scale. A greater number of communities implies more potential sources, and thus, increases the chances of an empty patch being colonised and a population establishing. The location of introduced communities also affects the colonisation of initially *empty* patches, but this effect depends on the landscape size. In our small 5‐patch landscape, the fastest recovery is achieved by introducing communities into multiple peripheral patches. However, in larger landscapes, it is more beneficial to introduce communities into central, highly connected patches. These central patches are important for linking the landscape, enabling movement across it, and ensuring metapopulation persistence (e.g., Cumming et al. 2022; Thompson et al. 2017; Gilarranz et al. 2015).

An experiment by Saade et al. (2022) also found that the recolonization dynamics following patch extinctions depend on the spatial distribution of the intact patches, although less so on their number. The dependence of the spread of individuals across the landscape on patch configuration has also been demonstrated by Rayfield et al. (2023) and Gilarranz et al. (2017) using experiments and models. Our simulation and experimental results indicate that the interaction between the number and location of introduced communities depends on the size of the landscape. In summary, we find that how many and where communities are introduced affects the dispersal pathways, and thus, the colonisation of the landscape.

#### 5.1.2 Recovery of Populated Patches

Yet, the number and location of introduced communities have little effect on the recovery of the populations in patches where they are introduced. Similarly, Altermatt et al. (2011) found in an experiment that local populations in undisturbed patches are unaffected by the emigration of individuals into disturbed patches. By decomposing the contributions of local community dynamics (intra‐ and inter‐specific effects) and dispersal (immigration and emigration), we find that population growth in the populated patches is driven by the former (see also Bird et al. 2024; Bullock et al. 2020). The relatively small contribution of dispersal to local community growth aligns with the lack of substantial effect of spatial configuration on the recovery in *populated* patches.

However, we note the timescale of our study is relatively short and the observed dynamics are transient. As hinted by the model results, the relative contribution of local processes and dispersal may be highly dynamic (Figures S15, S16). We postulate that, as local populations fluctuate, the rescue effect of immigration demonstrated by many previous studies (e.g., Liu and Vidal 2025; Li et al. 2023; Staddon et al. 2010; Holyoak 2000; Gonzalez et al. 1998) may become more prevalent (see Ives et al. 2004). Moreover, the contribution of dispersal has been shown to depend on dispersal rate (Zelnik et al. 2019; Thompson et al. 2017; Altermatt et al. 2011) and the dispersal kernel (Rayfield et al. 2023). This points to not only species specificity in the effects of spatial configuration on recovery, but also the influence of the landscape matrix (e.g., Fletcher et al. 2024; Aström and Pärt 2013).

### 5.2 The Role of Food‐Web Complexity

The complexity of the introduced communities has a profound effect on recovery at both local and regional scales. The recovery of our focal aphid species reduces upon the addition of another aphid species (i.e., interspecific competition), and/or parasitoid wasps (i.e., parasitism). By impeding population growth locally, these interspecific interactions reduce the overall dispersal potential and recovery at the landscape scale. However, there is empirical evidence that interspecific interactions affect dispersal and, in turn, colonisation in other ways. For example, both top‐down and bottom‐up control has been shown to increase emigration rates (Fronhofer et al. 2018), herbivores can alter dispersal distances of plants (Allbee et al. 2023), and detrimental versus beneficial interactions tend to promote or suppress dispersal, respectively (Bestion et al. 2024). In aphids, parasitoids can induce the production of winged morphs, leading to greater dispersal potential (Sloggett and Weisser 2002). In fact, our experimental results also suggest that dispersal may increase in the presence of parasitoids: recovery in initially *empty* patches is higher than predicted by our model, which does not include this mechanism (Figure 2A). Yet overall, this potential increase in dispersal is swamped by the negative effects of competition and parasitism on population growth (see also Bullock et al. 2020).

However, the negative effects of interspecific competition and parasitism could be, at least partially, offset by even greater food‐web complexity. For example, our model simulations suggest that the addition of a hyperparasitoid species (i.e., a higher trophic level) increases aphid's recovery by reducing the parasitism pressure, i.e., mesopredator suppression (Ritchie and Johnson 2009; May and Hassell 1981) or trophic cascade (Paine 1980) (but see Horn 1989 for evidence of more complex spatial dynamics at play). Alternatively, the addition of a third aphid species increases the focal aphid's recovery relative to the community with two aphid and two parasitoid species. This positive effect, despite another source of interspecific competition, is due to a reduced parasitism rate on each aphid species, that is, a dilution effect (Foster and Treherne 1981). In summary, higher food‐web complexity allows for more indirect effects among species, affecting recovery in less predictable ways.

### 5.3 Caveats and Future Directions

Here, we study ecological recovery from the perspective of a herbivore. Yet, different species and species guilds may be affected by space and community differently, and thus follow different recovery trajectories. This is perhaps most obvious in species involved in trophic interactions where the recovery of the resource benefits the consumer, but not vice versa (as shown here, also see Gawecka and Bascompte 2021). However, even among species belonging to the same guild, recovery depends on the number of interaction partners (Gawecka and Bascompte 2023). In species‐rich communities, patterns of interactions such as modularity may also exacerbate differences between species in their dispersal and colonisation success (Massol et al. 2017; Montoya et al. 2015). Additionally, species dispersal abilities can vary widely across trophic levels (Elzinga et al. 2007). This affects how species perceive the landscape (e.g., Bertellotti et al. 2023), and thus, the role of spatial configuration in recovery. In short, recovery trajectories of species at various trophic levels in species‐ and interaction‐rich communities remain to be investigated.

Our landscapes consist of equally sized patches with identical habitat. However, both habitat area and type influence species distributions (e.g., Ryser et al. 2024, 2021). Moreover, these effects can be species‐specific (e.g., Dong et al. 2025; Gardner et al. 2024; Twining et al. 2022; Van Noordwijk et al. 2015), and may influence interspecific interactions (Lennox et al. 2025). Incorporating heterogeneity in habitat quality or area could reveal new dynamics in recovery processes, and may be critical for scaling up our findings to more complex and realistic landscapes.

Our experimental and modelling framework simplifies the dispersal process relative to natural systems (Parry 2013; Woodford 1973). The physical connections between patches and the strict stepping‐stone configuration restrict movement to adjacent patches, excluding longer‐distance dispersal or patch‐skipping that may occur in the field. As such, our measured dispersal rates are likely to differ from those in natural systems. These simplifications were intentional: they allow us to model highly fragmented landscapes and to isolate the effect of spatial configuration under controlled conditions. Future work should test the generality of our findings by using more realistic dispersal kernels (Pleydell et al. 2018) and alternative landscape structures that account for patch isolation and the surrounding matrix.

## 6 Conclusions

There is a trade‐off between species recovery and food‐web complexity. Yet, we need complexity for ecosystem functioning and resilience (Liang et al. 2025; Tilman et al. 2014). We propose three approaches to this conundrum:

1. Spatial planning which considers landscape structure—introducing communities into multiple patches and/or prioritising highly connected patches for introductions may aid landscape colonisation,
2. Building species‐ and interaction‐rich communities—indirect effects within communities may boost the recovery relative to species‐poor communities,
3. Staggered species introductions—allowing lower trophic levels to establish before introducing higher trophic levels.

Our results suggest that ecological restoration involves a delicate balancing act. Despite this, much restoration practice takes little account of community or spatial complexities (Maes et al. 2024; Bullock et al. 2022). However, by integrating species interactions and spatial landscape configuration into restoration planning, it is likely that we can enhance recovery.

## Author Contributions

K.A.G. and J.B. conceived ideas, K.A.G., M.A.B. and J.B. designed methodology; K.A.G. conducted experiments and model simulations; K.A.G. and M.A.B. analysed data with input from J.M.B. and J.B.; K.A.G. wrote the first draft of the manuscript, and all authors contributed substantially to revisions.

## Peer Review

The peer review history for this article is available at https://www.webofscience.com/api/gateway/wos/peer‐review/10.1111/ele.70254.

## Supporting information


**Data S1:** ele70254‐sup‐0001‐Supinfo01.pdf.

## Data Availability

All data and code used in this study are available on GitHub (github.com/kgawecka/recovery_foodweb_experiment), and have been archived on Zenodo (zenodo.org/records/17404568, Gawecka et al. 2025).

## References

[ele70254-bib-0001] Agrawal, A. A. 2004. “Plant Defense and Density Dependence in the Population Growth of Herbivores.” American Naturalist 164, no. 1: 113–120. 10.1086/420980 15266375

[ele70254-bib-0002] Allbee, S. A. , H. S. Rogers , and L. L. Sullivan . 2023. “The Effects of Dispersal, Herbivory, and Competition on Plant Community Assembly.” Ecology 104, no. 1: e3859. 36054771 10.1002/ecy.3859 PMC10078099

[ele70254-bib-0003] Altermatt, F. , A. Bieger , F. Carrara , A. Rinaldo , and M. Holyoak . 2011. “Effects of Connectivity and Recurrent Local Disturbances on Community Structure and Population Density in Experimental Metacommunities.” PLoS One 6, no. 4: e19525. 21559336 10.1371/journal.pone.0019525 PMC3084878

[ele70254-bib-0004] Aoyama, L. , L. G. Shoemaker , B. Gilbert , et al. 2022. “Application of Modern Coexistence Theory to Rare Plant Restoration Provides Early Indication of Restoration Trajectories.” Ecological Applications 32, no. 7: e2649. 35560687 10.1002/eap.2649 PMC9787931

[ele70254-bib-0005] Arancibia, P. A. 2024. “The Topology of Spatial Networks Affects Stability in Experimental Metacommunities.” Proceedings of the Royal Society B: Biological Sciences 291, no. 2024: 1–8. 10.1098/rspb.2024.0567 PMC11338566 38864323

[ele70254-bib-0006] Arancibia, P. A. , and P. J. Morin . 2022. “Network Topology and Patch Connectivity Affect Dynamics in Experimental and Model Metapopulations.” Journal of Animal Ecology 91, no. 2: 496–505. 34873688 10.1111/1365-2656.13647

[ele70254-bib-0007] Aström, J. , and T. Pärt . 2013. “Negative and Matrix‐Dependent Effects of Dispersal Corridors in an Experimental Metacommunity.” Ecology 94, no. 1: 72–82. 23600242 10.1890/11-1795.1

[ele70254-bib-0008] Atkinson, J. , L. A. Brudvig , M. Mallen‐Cooper , S. Nakagawa , A. T. Moles , and S. P. Bonser . 2022. “Terrestrial Ecosystem Restoration Increases Biodiversity and Reduces Its Variability, but Not to Reference Levels: A Global Meta‐Analysis.” Ecology Letters 25, no. 7: 1725–1737. 35559594 10.1111/ele.14025 PMC9320827

[ele70254-bib-0009] Baker, C. M. , M. Bode , N. Dexter , et al. 2019. “A Novel Approach to Assessing the Ecosystem‐Wide Impacts of Reintroductions.” Ecological Applications 29, no. 1: e01811. 30312496 10.1002/eap.1811

[ele70254-bib-0010] Banin, L. F. , E. H. Raine , L. M. Rowland , et al. 2023. “The Road to Recovery: A Synthesis of Outcomes From Ecosystem Restoration in Tropical and Sub‐Tropical Asian Forests.” Philosophical Transactions of the Royal Society, B: Biological Sciences 378, no. 1867: 1–17. 10.1098/rstb.2021.0090 PMC9661948 36373930

[ele70254-bib-0011] Barbour, M. A. , D. J. Kliebenstein , and J. Bascompte . 2022. “A Keystone Gene Underlies the Persistence of an Experimental Food Web.” Science 376, no. 6588: 70–73. 35357912 10.1126/science.abf2232

[ele70254-bib-0012] Bastolla, U. , M. A. Fortuna , A. Pascual‐García , A. Ferrera , B. Luque , and J. Bascompte . 2009. “The Architecture of Mutualistic Networks Minimizes Competition and Increases Biodiversity.” Nature 458, no. 7241: 1018–1020. 19396144 10.1038/nature07950

[ele70254-bib-0013] Bertellotti, F. , N. R. Sommer , O. J. Schmitz , and M. A. Mccary . 2023. “Impacts of Habitat Connectivity on Grassland Arthropod Metacommunity Structure: A Field‐Based Experimental Test of Theory.” Ecology and Evolution 13, no. 11: e10686. 38020703 10.1002/ece3.10686 PMC10630154

[ele70254-bib-0014] Bestion, E. , D. Legrand , C. B. Baines , et al. 2024. “Species Interactions Affect Dispersal: A Meta‐Analysis.” Philosophical Transactions of the Royal Society, B: Biological Sciences 379, no. 1907: 1–18. 10.1098/rstb.2023.0127 PMC11391282 38913065

[ele70254-bib-0015] Bhandary, S. , K. A. Gawecka , F. Pedraza , and J. Bascompte . 2025. “Landscape Configuration and Community Structure Jointly Determine the Persistence of Mutualists Under Habitat Loss. *bioRxiv*.” 10.1098/rspb.2025.2602 42049218

[ele70254-bib-0016] Bird, J. P. , R. A. Fuller , and J. D. Shaw . 2024. “Patterns of Recovery in Extant and Extirpated Seabirds After the World's Largest Multipredator Eradication.” Conservation Biology 38, no. 4: e14239. 38375602 10.1111/cobi.14239

[ele70254-bib-0017] Bowler, D. E. , and T. G. Benton . 2005. “Causes and Consequences of Animal Dispersal Strategies: Relating Individual Behaviour to Spatial Dynamics.” Biological Reviews 80, no. 2: 205–225. 15921049 10.1017/s1464793104006645

[ele70254-bib-0018] Bowler, D. E. , and T. G. Benton . 2009. “Impact of Dispersal on Population Growth: The Role of Inter‐Patch Distance.” Oikos 118, no. 3: 403–412.

[ele70254-bib-0019] Brudvig, L. A. 2011. “The Restoration of Biodiversity: Where Has Research Been and Where Does It Need to Go?” American Journal of Botany 98, no. 3: 549–558. 21613146 10.3732/ajb.1000285

[ele70254-bib-0020] Bullock, J. M. , E. Fuentes‐Montemayor , B. Mccarthy , et al. 2022. “Future Restoration Should Enhance Ecological Complexity and Emergent Properties at Multiple Scales.” Ecography 2022, no. 4: 1–11.

[ele70254-bib-0021] Bullock, J. M. , I. L. Moy , R. F. Pywell , S. J. Coulson , A. M. Nolan , and H. Caswell . 2002. “Plant Dispercsal and Colonization Processes at Local and Landscape Scales.” In Dispersal Ecology, edited by J. M. Bullock , R. E. Kenward , and R. S. Hails . Blackwell Publishing.

[ele70254-bib-0022] Bullock, J. M. , M. C. Wichmann , R. S. Hails , et al. 2020. “Human‐Mediated Dispersal and Disturbance Shape the Metapopulation Dynamics of a Long‐Lived Herb.” Ecology 101, no. 8: e03087. 32320472 10.1002/ecy.3087

[ele70254-bib-0023] Cumming, G. S. , R. A. Magris , and K. Maciejewski . 2022. “Quantifying Cross‐Scale Patch Contributions to Spatial Connectivity.” Landscape Ecology 37, no. 9: 2255–2272.

[ele70254-bib-0024] Domínguez‐Garcia, V. , F. P. Molina , O. Godoy , and I. Bartomeus . 2024. “Interaction Network Structure Explains Species' Temporal Persistence in Empirical Plant–Pollinator Communities.” Nature Ecology & Evolution 8, no. 3: 423–429. 38302580 10.1038/s41559-023-02314-3

[ele70254-bib-0025] Dong, Z. , A. J. Bladon , C. C. Jaworski , et al. 2025. “Species‐Habitat Networks Reveal Conservation Implications That Other Community Analyses Do Not Detect.” Ecological Applications 35, no. 1: e3073. 39829221 10.1002/eap.3073 PMC11744225

[ele70254-bib-0026] Elzinga, J. A. , S. Van Nouhuys , D.‐J. Van Leeuwen , and A. Biere . 2007. “Distribution and Colonisation Ability of Three Parasitoids and Their Herbivorous Host in a Fragmented Landscape.” Basic and Applied Ecology 8, no. 1: 75–88.

[ele70254-bib-0027] Escobar, S. , F. L. Newell , M. J. Endara , et al. 2025. “Reassembly of a Tropical Rainforest: A New Chronosequence in the Chocó Tested With the Recovery of Tree Attributes.” Ecosphere 16, no. 2: e70157.

[ele70254-bib-0028] Fernandez‐Triana, J. , M. R. Shaw , C. Boudreault , M. Beaudin , and G. R. Broad . 2020. “Annotated and Illustrated World Checklist of Microgastrinae Parasitoid Wasps (Hymenoptera, Braconidae).” ZooKeys 920: 1–1089. 32390740 10.3897/zookeys.920.39128 PMC7197271

[ele70254-bib-0029] Finke, D. L. , and W. E. Snyder . 2008. “Niche Partitioning Increases Resource Exploitation by Diverse Communities.” Science 321, no. 5895: 1488–1490. 18787167 10.1126/science.1160854

[ele70254-bib-0030] Firkowski, C. R. , P. L. Thompson , A. Gonzalez , M. W. Cadotte , and M. J. Fortin . 2022. “Multi‐Trophic Metacommunity Interactions Mediate Asynchrony and Stability in Fluctuating Environments.” Ecological Monographs 92, no. 1: e01484.

[ele70254-bib-0031] Fletcher, R. J. , T. Smith , S. Troy , et al. 2024. “The Prominent Role of the Matrix in Ecology, Evolution, and Conservation.” Annual Review of Ecology, Evolution, and Systematics 55, no. 1: 423–447.

[ele70254-bib-0032] Foster, W. A. , and J. E. Treherne . 1981. “Evidence for the Dilution Effect in the Selfish Herd From Fish Predation on a Marine Insect.” Nature 293, no. 5832: 466–467.

[ele70254-bib-0033] Fronhofer, E. A. , D. Legrand , F. Altermatt , et al. 2018. “Bottom‐Up and Top‐Down Control of Dispersal Across Major Organismal Groups.” Nature Ecology & Evolution 2, no. 12: 1859–1863. 30397298 10.1038/s41559-018-0686-0

[ele70254-bib-0034] Gaiarsa, M. P. , and J. Bascompte . 2022. “Hidden Effects of Habitat Restoration on the Persistence of Pollination Networks.” Ecology Letters 25, no. 10: 2132–2141. 36006740 10.1111/ele.14081 PMC9804604

[ele70254-bib-0035] Gardner, E. , R. A. Robinson , A. Julian , et al. 2024. “A Family of Process‐Based Models to Simulate Landscape Use by Multiple Taxa.” Landscape Ecology 39, no. 5: 1–26.

[ele70254-bib-0036] Gawecka, K. A. , M. A. Barbour , J. M. Bullock , and J. Bascompte . 2025. “Data From: The Roles of Space and Food‐Web Complexity in Mediating Ecological Recovery. Zenodo.” 10.5281/zenodo.17404568.41248218

[ele70254-bib-0037] Gawecka, K. A. , and J. Bascompte . 2021. “Habitat Restoration in Spatially Explicit Metacommunity Models.” Journal of Animal Ecology 90, no. 5: 1239–1251. 33630316 10.1111/1365-2656.13450

[ele70254-bib-0038] Gawecka, K. A. , and J. Bascompte . 2023. “Habitat Restoration and the Recovery of Metacommunities.” Journal of Applied Ecology 60, no. 8: 1622–1636.

[ele70254-bib-0039] Gilarranz, L. J. , and J. Bascompte . 2012. “Spatial Network Structure and Metapopulation Persistence.” Journal of Theoretical Biology 297, no. 2012: 11–16. 22155351 10.1016/j.jtbi.2011.11.027

[ele70254-bib-0040] Gilarranz, L. J. , B. Rayfield , G. Liñán‐Cembrano , J. Bascompte , and A. Gonzalez . 2017. “Effects of Network Modularity on the Spread of Perturbation Impact in Experimental Metapopulations.” Science 357, no. 6347: 199–201. 28706071 10.1126/science.aal4122

[ele70254-bib-0041] Gilarranz, L. J. , M. Sabatino , M. A. Aizen , and J. Bascompte . 2015. “Hot Spots of Mutualistic Networks.” Journal of Animal Ecology 84, no. 2: 407–413. 25402941 10.1111/1365-2656.12304

[ele70254-bib-0042] Gonzalez, A. , J. H. Lawton , F. S. Gilbert , T. M. Blackburn , and I. Evans‐Freke . 1998. “Metapopulation Dynamics, Abundance, and Distribution in a Microecosystem.” Science 281, no. 5385: 2045–2047. 9748167 10.1126/science.281.5385.2045

[ele70254-bib-0043] Hallett, L. M. , L. Aoyama , G. Barabás , et al. 2023. “Restoration Ecology Through the Lens of Coexistence Theory.” Trends in Ecology & Evolution 38, no. 11: 1085–1096. 37468343 10.1016/j.tree.2023.06.004

[ele70254-bib-0044] Hanski, I. 2000. “Extinction Debt and Species Credit in Boreal Forests: Modelling the Consequences of Different Approaches to Biodiversity Conservation.” Annales Zoologici Fennici 37, no. 4: 271–280.

[ele70254-bib-0045] Holyoak, M. 2000. “Habitat Patch Arrangement and Metapopulation Persistence of Predators and Prey.” American Naturalist 156, no. 4: 378–389.

[ele70254-bib-0046] Hordijk, I. , L. Poorter , J. A. Meave , et al. 2024. “Land Use History and Landscape Forest Cover Determine Tropical Forest Recovery.” Journal of Applied Ecology 61, no. 10: 2365–2381.

[ele70254-bib-0047] Horn, D. J. 1989. “Secondary Parasitism and Population Dynamics of Aphid Parasitoids (Hymenoptera: Aphidiidae).” Journal of the Kansas Entomological Society 62, no. 2: 203–210.

[ele70254-bib-0048] Horn, S. , M. Coll , H. Asmus , and T. Dolch . 2020. “Food Web Models Reveal Potential Ecosystem Effects of Seagrass Recovery in the Northern Wadden Sea.” Restoration Ecology 29, no. S2: e13328.

[ele70254-bib-0049] Isaac, N. J. B. , P. N. M. Brotherton , J. M. Bullock , et al. 2018. “Defining and Delivering Resilient Ecological Networks: Nature Conservation in England.” Journal of Applied Ecology 55, no. 6: 2537–2543.

[ele70254-bib-0050] Ives, A. R. , S. T. Woody , E. V. Nordheim , C. Nelson , and J. H. Andrews . 2004. “The Synergistic Effects of Stochasticity and Dispersal on Population Densities.” American Naturalist 163, no. 3: 375–387. 10.1086/381942 15026974

[ele70254-bib-0051] Leclère, D. , M. Obersteiner , M. Barrett , et al. 2020. “Bending the Curve of Terrestrial Biodiversity Needs an Integrated Strategy.” Nature 585, no. 7826: 551–556. 32908312 10.1038/s41586-020-2705-y

[ele70254-bib-0052] Leibold, M. A. , M. Holyoak , N. Mouquet , et al. 2004. “The Metacommunity Concept: A Framework for Multi‐Scale Community Ecology.” Ecology Letters 7, no. 7: 601–613.

[ele70254-bib-0053] Lennox, R. J. , M. Kambestad , S. Berhe , et al. 2025. “The Role of Habitat in Predator–Prey Dynamics With Applications to Restoration.” Restoration Ecology 33, no. 3: e14354.

[ele70254-bib-0054] Levins, R. 1966. “The Strategy of Model Building in Population Biology.” American Scientist 54, no. 4: 421–431.

[ele70254-bib-0055] Li, D. , J. Memmott , and C. F. Clements . 2023. “Corridor Quality Buffers Extinction Under Extreme Droughts in Experimental Metapopulations.” Ecology and Evolution 13, no. 6: e10166. 37274153 10.1002/ece3.10166 PMC10234780

[ele70254-bib-0056] Liang, M. , Q. Yang , J. M. Chase , et al. 2025. “Unifying Spatial Scaling Laws of Biodiversity and Ecosystem Stability.” Science 387, no. 6740: 1–8. 10.1126/science.adl2373 40112067

[ele70254-bib-0057] Liu, C. , and M. C. Vidal . 2025. “Dispersal Promotes Stability and Persistence of Exploited Yeast Mutualisms.” ISME Journal 19, no. 1: 1–9. 10.1093/ismejo/wraf003 PMC11778857 39787040

[ele70254-bib-0058] Maes, S. L. , M. P. Perring , R. Cohen , et al. 2024. “Explore Before You Restore: Incorporating Complex Systems Thinking in Ecosystem Restoration.” Journal of Applied Ecology 61, no. 5: 922–939.

[ele70254-bib-0059] Marjakangas, E. L. , L. Genes , M. M. Pires , and F. Fernandez . 2018. “Estimating Interaction Credit for Trophic Rewilding in Tropical Forests.” Philosophical Transactions of the Royal Society, B: Biological Sciences 373, no. 1761: 1–8. 10.1098/rstb.2017.0435 PMC6231069 30348879

[ele70254-bib-0060] Massol, F. , M. Dubart , V. Calcagno , et al. 2017. Island Biogeography of Food Webs. Elsevier.

[ele70254-bib-0061] May, R. M. , and M. P. Hassell . 1981. “The Dynamics of Multiparasitoid‐Host Interactions.” American Naturalist 117, no. 3: 234–261.

[ele70254-bib-0062] Minor, E. S. , and D. L. Urban . 2008. “A Graph‐Theory Framework for Evaluating Landscape Connectivity and Conservation Planning.” Conservation Biology 22, no. 2: 297–307. 18241238 10.1111/j.1523-1739.2007.00871.x

[ele70254-bib-0063] Montoya, D. 2021. “Challenges and Directions Toward a General Theory of Ecological Recovery Dynamics: A Metacommunity Perspective.” One Earth 4, no. 8: 1083–1094.

[ele70254-bib-0064] Montoya, D. , M. L. Yallop , and J. Memmott . 2015. “Functional Group Diversity Increases With Modularity in Complex Food Webs.” Nature Communications 6, no. 1: 7379. 10.1038/ncomms8379 PMC4490355 26059871

[ele70254-bib-0065] Moreno‐Mateos, D. , E. B. Barbier , P. C. Jones , et al. 2017. “Anthropogenic Ecosystem Disturbance and the Recovery Debt.” Nature Communications 8, no. 1: 14163. 10.1038/ncomms14163 PMC5263871 28106039

[ele70254-bib-0066] Oliver, T. H. , M. S. Heard , N. J. B. Isaac , et al. 2015. “Biodiversity and Resilience of Ecosystem Functions.” Trends in Ecology & Evolution 30, no. 11: 673–684. 26437633 10.1016/j.tree.2015.08.009

[ele70254-bib-0067] Paine, R. T. 1980. “Food Webs: Linkage, Interaction Strength and Community Infrastructure.” Journal of Animal Ecology 49, no. 3: 667–685.

[ele70254-bib-0068] Parry, H. R. 2013. “Cereal Aphid Movement: General Principles and Simulation Modelling.” Movement Ecology 1, no. 1: 1–14. 25709827 10.1186/2051-3933-1-14 PMC4337770

[ele70254-bib-0069] Pettorelli, N. , and J. M. Bullock . 2023. “Restore or Rewild? Implementing Complementary Approaches to Bend the Curve on Biodiversity Loss.” Ecological Solutions and Evidence 4, no. 2: e12244.

[ele70254-bib-0070] Pleydell, D. R. J. , S. Soubeyrand , S. Dallot , et al. 2018. “Estimation of the Dispersal Distances of an Aphid‐Borne Virus in a Patchy Landscape.” PLoS Computational Biology 14, no. 4: e1006085. 29708968 10.1371/journal.pcbi.1006085 PMC5945227

[ele70254-bib-0071] Prima, M. C. , T. Duchesne , A. Fortin , L. P. Rivest , and D. Fortin . 2018. “Combining Network Theory and Reaction–Advection–Diffusion Modelling for Predicting Animal Distribution in Dynamic Environments.” Methods in Ecology and Evolution 9, no. 5: 1221–1231.

[ele70254-bib-0083] R Core Team . 2020. R: A Language and Environment for Statistical Computing. Austria.

[ele70254-bib-0072] Rayfield, B. , C. B. Baines , L. J. Gilarranz , and A. Gonzalez . 2023. “Spread of Networked Populations Is Determined by the Interplay Between Dispersal Behavior and Habitat Configuration.” Proceedings of the National Academy of Sciences 120, no. 11: e2201553120. 10.1073/pnas.2201553120 PMC10089154 36893275

[ele70254-bib-0073] Resch, M. C. , M. Schütz , R. Ochoa‐Hueso , et al. 2022. “Long‐Term Recovery of Above‐ and Below‐Ground Interactions in Restored Grasslands After Topsoil Removal and Seed Addition.” Journal of Applied Ecology 59, no. 9: 2299–2308.

[ele70254-bib-0074] Ritchie, E. G. , and C. N. Johnson . 2009. “Predator Interactions, Mesopredator Release and Biodiversity Conservation.” Ecology Letters 12, no. 9: 982–998. 19614756 10.1111/j.1461-0248.2009.01347.x

[ele70254-bib-0075] Rohr, R. P. , S. Saavedra , and J. Bascompte . 2014. “On the Structural Stability of Mutualistic Systems.” Science 345, no. 6195: 1253497. 25061214 10.1126/science.1253497

[ele70254-bib-0076] Ryser, R. , J. M. Chase , B. Gauzens , et al. 2024. “Landscape Configuration Can Flip Species–Area Relationships in Dynamic Meta‐Food‐Webs.” Philosophical Transactions of the Royal Society, B: Biological Sciences 379, no. 1907: 1–9. 10.1098/rstb.2023.0138 PMC11391306 38913064

[ele70254-bib-0077] Ryser, R. , M. R. Hirt , J. Häussler , D. Gravel , and U. Brose . 2021. “Landscape Heterogeneity Buffers Biodiversity of Simulated Meta‐Food‐Webs Under Global Change Through Rescue and Drainage Effects.” Nature Communications 12, no. 1: 4716. 10.1038/s41467-021-24877-0 PMC8342463 34354058

[ele70254-bib-0078] Saade, C. , S. Kefi , C. Gougat‐Barbera , B. Rosenbaum , and E. A. Fronhofer . 2022. “Spatial Autocorrelation of Local Patch Extinctions Drives Recovery Dynamics in Metacommunities.” Proceedings of the Royal Society B: Biological Sciences 289, no. 1972: 1–10. 10.1098/rspb.2022.0543 PMC9006024 35414238

[ele70254-bib-0079] Sloggett, J. J. , and W. W. Weisser . 2002. “Parasitoids Induce Production of the Dispersal Morph of the Pea Aphid, *Acyrthosiphon pisum* .” Oikos 98, no. 2: 323–333.

[ele70254-bib-0080] Staddon, P. , Z. Lindo , P. D. Crittenden , F. Gilbert , and A. Gonzalez . 2010. “Connectivity, Non‐Random Extinction and Ecosystem Function in Experimental Metacommunities.” Ecology Letters 13, no. 5: 543–552. 20236160 10.1111/j.1461-0248.2010.01450.x

[ele70254-bib-0081] Suding, K. N. 2011. “Toward an Era of Restoration in Ecology: Successes, Failures, and Opportunities Ahead.” Annual Review of Ecology, Evolution, and Systematics 42, no. 1: 465–487.

[ele70254-bib-0082] Sutheimer, C. M. , A. T. Filicetti , L. Viliani , and S. E. Nielsen . 2025. “Regeneration Lags and Growth Trajectories Influence Passive Seismic Line Recovery in Western North American Boreal Forests.” Restoration Ecology 33, no. 3: e14353.

[ele70254-bib-0084] Thébault, E. , and C. Fontaine . 2010. “Stability of Ecological Communities and the Architecture of Mutualistic and Trophic Networks.” Science 329, no. 5993: 853–856. 20705861 10.1126/science.1188321

[ele70254-bib-0085] Thompson, P. L. , and A. Gonzalez . 2017. “Dispersal Governs the Reorganization of Ecological Networks Under Environmental Change.” Nature Ecology & Evolution 1, no. 6: 162. 28812626 10.1038/s41559-017-0162

[ele70254-bib-0086] Thompson, P. L. , B. Rayfield , and A. Gonzalez . 2017. “Loss of Habitat and Connectivity Erodes Species Diversity, Ecosystem Functioning, and Stability in Metacommunity Networks.” Ecography 40, no. 1: 98–108.

[ele70254-bib-0087] Tilman, D. , F. Isbell , and J. M. Cowles . 2014. “Biodiversity and Ecosystem Functioning.” Annual Review of Ecology, Evolution, and Systematics 45, no. 1: 471–493.

[ele70254-bib-0088] Twining, J. P. , C. Sutherland , N. Reid , and D. G. Tosh . 2022. “Habitat Mediates Coevolved but Not Novel Species Interactions.” Proceedings of the Royal Society B: Biological Sciences 289, no. 1966: 1–9. 10.1098/rspb.2021.2338 PMC8753165 35016538

[ele70254-bib-0089] Tylianakis, J. M. , E. Laliberté , A. Nielsen , and J. Bascompte . 2010. “Conservation of Species Interaction Networks.” Biological Conservation 143, no. 10: 2270–2279.

[ele70254-bib-0090] Van Noordwijk, C. G. E. , W. C. E. P. Verberk , H. Turin , et al. 2015. “Species–Area Relationships Are Modulated by Trophic Rank, Habitat Affinity, and Dispersal Ability.” Ecology 96, no. 2: 518–531. 26240873 10.1890/14-0082.1

[ele70254-bib-0091] Van Nouhuys, S. , and I. Hanski . 2002. “Colonization Rates and Distances of a Host Butterfly and Two Specific Parasitoids in a Fragmented Landscape.” Journal of Animal Ecology 71, no. 4: 639–650.

[ele70254-bib-0092] Woodford, J. 1973. “The Flight Activity and Movements of *Myzus persicae* (Sulzer) and *Brevicoryne brassicae* (l.) in a Field Cage.” Journal of Applied Ecology 10, no. 3: 803–824.

[ele70254-bib-0093] Zelnik, Y. R. , J.‐F. Arnoldi , and M. Loreau . 2019. “The Three Regimes of Spatial Recovery.” Ecology 100, no. 2: e02586. 30556129 10.1002/ecy.2586 PMC6375383

