# Supplementary material for: The Roles of Space and Food‐Web Complexity in Mediating Ecological Recovery
Source: Ecol Lett. 2025 Nov 17;28(11):e70254. doi: 10.1111/ele.70254 (PMC12622866; doi:10.1111/ele.70254)
Supplement: Supplementary file 1 — Data S1: ele70254‐sup‐0001‐Supinfo01.pdf. [file ELE-28-0-s001.pdf]

Supporting Information

The roles of space and food-web complexity in mediating ecological recovery

Klementyna A. Gawecka[1,2*], Matthew A. Barbour[3], James M. Bullock[4] & Jordi Bascompte[1]

[1] Department of Evolutionary Biology and Environmental Studies, University of Zurich, Winterthurerstrasse 190, 8057 Zürich, Switzerland
[2] UK Centre for Ecology & Hydrology, Bush Estate, Penicuik, EH26 0QB, UK (current affiliation)
[3] Département de biologie, Université de Sherbrooke, 2500 boulevard de l'Université, J1K 2R1 Sherbrooke, Québec, Canada
[4] UK Centre for Ecology & Hydrology, Benson Lane, Crowmarsh Gifford, Wallingford, OX10 8BB, UK

* klementyna.gawecka@gmail.com

Contents

Metacommunity model ... 3
- Description ... 3
- Parameterisation ... 5
  - Single aphid species response experiment ... 5
  - Two aphid species response experiment ... 7
  - Maximum parasitisation experiment ... 7
  - Parasitoid functional response experiment ... 8
  - Time to emergence experiment ... 8
  - Fraction of female parasitoids experiment ... 9
  - Parasitoid lifespan experiment ... 9
  - Parasitoid dispersal experiment ... 9
  - Non-experimental insect species ... 12

Experimental landscapes and communities ... 14
- Experimental results ... 14
- Model simulations ... 19

Larger landscapes and communities ... 25

References ... 28

Metacommunity model

Description

We developed a metacommunity dynamics model describing the local and spatial dynamics of our insects. To facilitate comparison with experimental data and parameterisation, we adopted a discrete-time model, with a timestep of one day. Prior to the main experiment, we carried out a series of experiments designed to (1) inform the choice of mathematical functions in the model and (2) determine model parameters (see Parameterisation section below).

The population size of aphid species i in patch k at time t is given by:

$$A_{i,k}^{t+1} = G_{i,k}^{t} - M_{i,k}^{t} - E_{i,k}^{t} + I_{i,k}^{t} \qquad \text{Eq. S 1}$$

The first term ($G_{i,k}^{t}$) includes the intrinsic growth, and intra- and inter-specific competition, and is given by:

$$G_{i,k}^{t} = A_{i,k}^{t} exp(r_i - \sum_{j=1}^{n} \alpha_{ij} A_{j,k}^{t}) \qquad \text{Eq. S 2}$$

where r_i is the intrinsic growth rate, α_{ij} is the intraspecific (when $j = i$) or interspecific competition (when $j \neq i$), and n is the number of aphid species. We considered several alternative mathematical formulations, including Ricker, Beverton-Holt and Gompertz models, for this term. We found that this exponential form of the logistic growth model (e.g., Agrawal, 2004) best reproduced the experimental data (Figure S 1, Figure S 3, Figure S 6).

The second term in Eq. S 1 ($M_{i,k}^{t}$) represents mortality due to parasitism. Based on the experimental observations (Figure S 4), we modelled it as a Type I functional response with a threshold for maximum number of parasitised aphids, M_{max} (thus mimicking a Type II response):

$$M_{i,k}^{t} = \begin{cases} \sum_{j=1}^{m} \beta_{ij} A_{i,k}^{t} P_{j,k}^{t} & if \quad \sum_{i=1}^{n} M_{i,k}^{t} \leq \frac{M_{max}}{\lambda} P_{j,k}^{t} \\ \sum_{j=1}^{m} c_{ij} \frac{M_{max}}{\lambda} P_{j,k}^{t} & otherwise \end{cases} \qquad \text{Eq. S 3}$$

where β_{ij} is the parasitoid's j attack rate on aphid i, P_k^{t-1} is the parasitoid population size (in terms of the number of females only), m is the number of parasitoid species, and λ is parasitoid's lifespan (in days). M_{max} represents the total number of parasitised aphids

throughout parasitoid's lifetime. Although the parasitisation rate has been shown to reduce throughout parasitoid's lifetime (Soni and Kumar, 2021), we assume a constant rate for simplicity. If this per-timestep quota is exceeded, we weigh the number of parasitised aphids of each species by $c_{ij} = \beta_{ij} A_{i,k}^t P_{j,k}^t / \sum_{i=1}^n M_{i,k}^t$. This ensures that the differences between aphids, in terms of their abundances and parasitoid attack rates, are accounted for.

The third term in Eq. S 1 ($E_{i,k}^t$) models the density-dependent aphid emigration from patch k (Figure S 2) and is given by:

$$E_{i,k}^t = \begin{cases} 0 & if \quad A_{i,k}^t < N_{min,i} \\ e_i(A_{i,k}^t - N_{min,i}) & if \quad A_{i,k}^t \geq N_{min,i} \end{cases} \qquad \text{Eq. S 4}$$

where $N_{min,i}$ is the minimum population size for emigration (also observed in other studies, e.g., Hodgson, 1991) and e_i is the emigration rate.

The final term in Eq. S 1 ($I_{i,k}^t$) represents immigration of aphids into patch k from adjacent patches and id given by:

$$I_{i,k}^t = \sum_{l=1}^{q} \frac{E_{i,l}^t}{c_l} \qquad \text{Eq. S 5}$$

where q is the number of directly connected patches to patch k, and c_l is the number of their direct connections.

We modelled the population size of the parasitoid j in patch k as:

$$P_{j,k}^{t+1} = P_{j,k}^t + B_{j,k}^t - D_{j,k}^t - E_{j,k}^t + I_{j,k}^t \qquad \text{Eq. S 6}$$

where emigration ($E_{j,k}^t$) and immigration ($I_{j,k}^t$) expressions have the same form as those for aphids (Eq. S 4 and Eq. S 5, Figure S 5). $B_{j,k}^t$ is the number of births defined as:

$$B_{j,k}^t = \begin{cases} 0 & if \quad t \leq \tau \\ f \sum_{i=1}^{n} M_{i,k}^{t-\tau} - \widehat{M}_{i,k}^{t-\tau} & if \quad t > \tau \end{cases} \qquad \text{Eq. S 7}$$

where f is the fraction of female parasitoids, τ is the time between parasitisation of an aphid and emergence of an adult parasitoid, $M_{i,k}^{t-\tau}$ (Eq. S 3) is the number of aphids parasitised at time $t - \tau$, and $\widehat{M}_{i,k}^{t-\tau}$ is the number of parasitoid larvae parasitised by the hyperparasitoid at time $t - \tau$. $\widehat{M}_{i,k}^{t-\tau}$ has the same form as Eq. S 3 but is a function of the number of parasitised aphids and hyperparasitoid density.

The number of parasitoid deaths, $D_{j,k}^{t}$, is given by:

$$D_{j,k}^{t} = \begin{cases} P_{j,k}^{t} & if \quad t = \lambda \\ \frac{P_{j,k}^{t}}{\sum_{k=1}^{p} P_{j,k}^{t}} \sum_{k=1}^{p} B_{j,k}^{t-\lambda} & if \quad t > \tau + \lambda \\ 0 & otherwise \end{cases} \quad \text{Eq. S 8}$$

The first expression ensures that initially placed parasitoid die at time $t = \lambda$. The second expression represents deaths of the emerged parasitoids. It weighs the total number of deaths across all p patches ($\sum_{k=1}^{p} B_{j,k}^{t-\lambda}$) by the number of parasitoids in patch k relative to the total number of parasitoids. This accounts for movement of individuals between patches.

The population size of the hyperparasitoid was modelled in the same way as the parasitoid (Eq. S 6-Eq. S 8).

Parameterisation

We conducted all parameterisation experiments in the same climate chamber (22 °C, 50 % humidity and 16 h photoperiod) and the same containers (Figure 1B) as in the main experiment. We used the same radish plant species (*Raphanus sativus*), aphid (*Brevicoryne brassicae* and *Lipaphis erysimi*) and parasitoid (*Diaeretiella rapae*) colonies as in the main experiment. In all cases, we used a single, two-week old radish plants per pot. When selecting aphid individuals, we ensured that all are adults of similar size (and age). The parasitoid wasps were maintained on a non-experimental aphid species (green peach aphid, *Myzus persicae*), and supplemented with honey solution throughout the experiments.

Single aphid species response experiment

Parameters determined: r_i, α_{ii}, e_i, $N_{min,i}$

Experimental procedure: We connected two patches and placed a radish plant in each patch. We placed 5 aphids of the same species (*B. brassicae* or *L. erysimi*) onto the radish leaves in one of the patches (patch 1). We ran the experiment for 28 days. Three days per week (Mondays, Wednesdays and Fridays), we counted aphids in both patches, and then removed all aphids present in the initially empty patch (patch 2). This allowed us to determine the population growth in patch 1, and emigration into patch 2. We replicated this setup five times per aphid species.

Parameter determination: We determined the per capita growth rate, accounting for emigrated aphids, E_i^{t-1}, and number of days between counts, Δt. We plotted it against population size (A_i^{t-1}) and fitted a linear model (Figure S 1). We found that the linear model fitted the experimental data best when we defined the per capita growth rate as: $\frac{\ln(A_i^t + E_i^{t-1}) - \ln(A_i^{t-1})}{\Delta t}$ (Agrawal, 2004). This results in the exponential expression given by Eq. S 2. We extracted the 95% confidence intervals of the intercept (i.e., r_i) and slope (i.e. α_{ii}). The

minimum population size for emigration, $N_{min,i}$, was the aphid count in patch 1 when we found aphids in patch 2 for the first time. For each aphid species, we took the average value across all replicas. To determine the emigration rate, we plotted the number of emigrated aphids per day against aphid count in patch 1, and fitted a linear model with $N_{min,i}$ as the x-axis intercept (Figure S 2). We then obtained the 95% confidence interval of the slope as e_i.

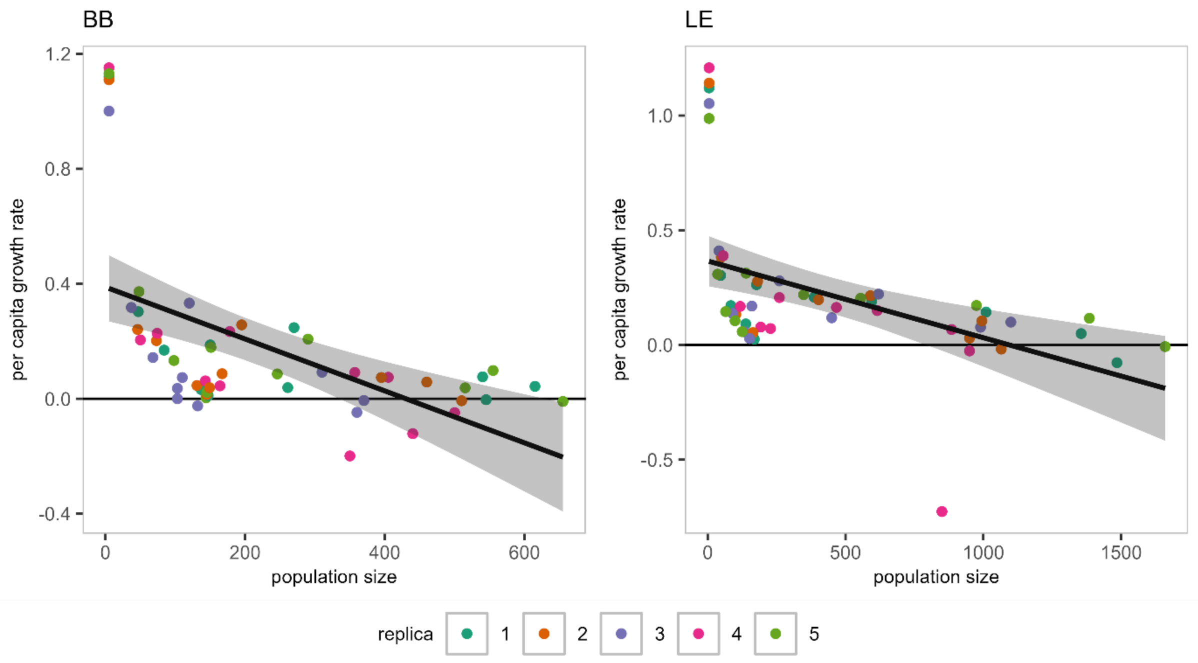

Figure S 1. Single aphid species response experiment: per capita growth rate vs population size (BB - *B. brassicae*, LE - *L. erysimi*).

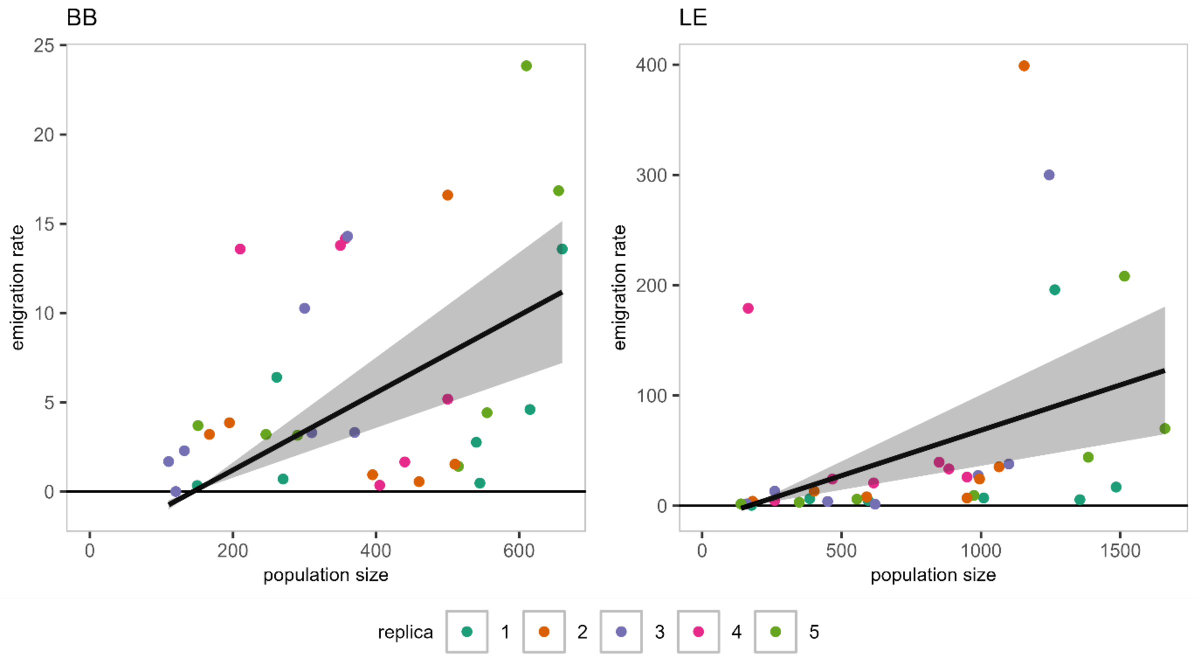

Figure S 2. Single aphid species response experiment: emigration rate vs population size (BB - *B. brassicae*, LE - *L. erysimi*).

Two aphid species response experiment

Parameters determined: α_{ij}

Experimental procedure: In a single container, we placed a radish plant and transferred 5 *B. brassicae* and 5 *L. erysimi* aphids. We ran the experiment for 28 days, counting the aphids three times per week (Mondays, Wednesdays, Fridays). We replicated the experiment five times.

Parameter determination: To determine interspecific competition, we plotted $r_i - \alpha_{ii} A_i^{t-1} - \frac{\ln(A_i^t + E_i^{t-1}) - \ln(A_i^{t-1})}{\Delta t}$ against A_j^{t-1} (Figure S 3). For r_i and α_{ii}, we substituted the estimates determined from the single aphid species response experiments (see above). We fitted a linear model with the intercept at the origin and extracted the 95% confidence interval of the slope as α_{ij}.

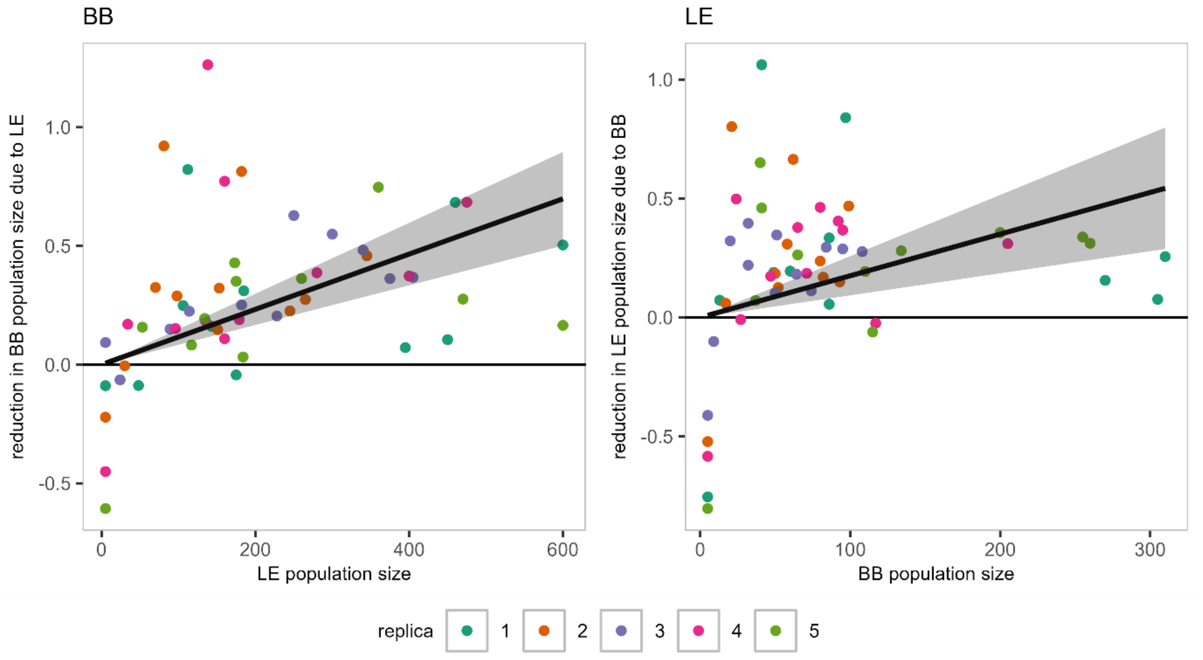

Figure S 3. Two aphid species response experiment: reduction in population size due to interspecific competition vs population size of competing aphid (BB - *B. brassicae*, LE - *L. erysimi*).

Maximum parasitisation experiment

Parameters determined: M_{max}

Experimental procedure: In a patch, we placed a radish plant and transferred 64 aphids of the same species (*B. brassicae* or *L. erysimi*) onto plant's leaves. We added a single one-day old female parasitoid. We maintained the container until mummies could be identified (i.e., beyond parasitoid's lifetime), at which point we counted them to determine the total number of parasitised aphids per parasitoid. We replicated the experiment five times per aphid species.

Parameter determination: As there was no substantial difference in the total number of parasitised aphids between the two aphid species, we determined M_{max} as the average mummy count across both aphid species and all replicas.

Parasitoid functional response experiment

Parameters determined: β_{ij}

Experimental procedure: We placed a radish plant in a single patch and transfer 4, 8, 16, 32 or 64 aphids of the same species (*B. brassicae* or *L. erysimi*). We added a single one-day old female parasitoid. After 24 hours, we removed the parasitoid. We maintained the patches in constant conditions until the mummies could be identified. We replicated each aphid density five times. We performed separate experiments involving the two aphid species. While is approach is suitable for food webs consisting of only one aphid and one parasitoid species, it may be oversimplified for larger communities with multiple aphid and/or parasitoid species (Rosenbaum et al., 2024).

Parameter determination: For each aphid species, we plotted the number of mummies against the initial number of aphids (Figure S 4). We fitted a linear model with zero intercept and obtained the 95% confidence interval of the slope as β_{ij}.

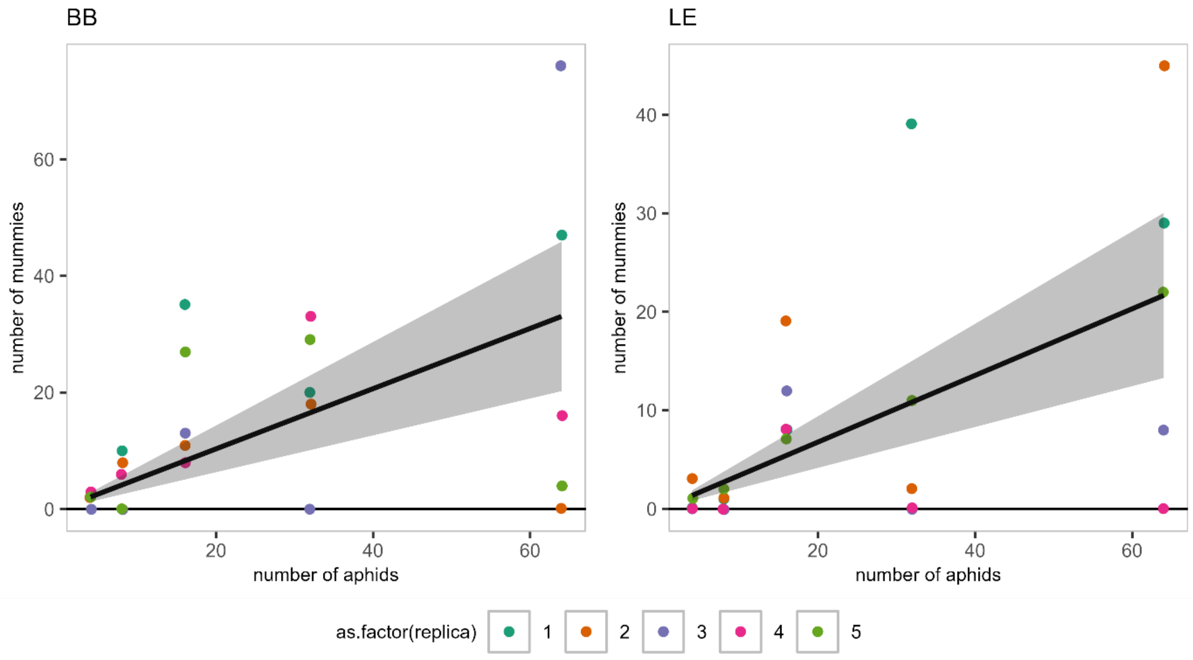

Figure S 4. Parasitoid functional response experiment: number of parasitoid mummies vs initial number of aphids (BB - *B. brassicae*, LE - *L. erysimi*).

Time to emergence experiment

Parameters determined: τ

Experimental procedure: This experiment was a continuation of the functional response experiment. Every day, we counted the number of emerged parasitoids and removed them from the patch.

Parameter determination: We determined the time to emergence as the mean number of days since parasitism, weighted by the number of emerged parasitoids that day. Due to lack of clear difference between the two aphid species, we averaged τ across *B. brassicae* and *L. erysimi*.

Fraction of female parasitoids experiment

Parameters determined: f

Experimental procedure: We collected parasitoids from a colony of newly emerged parasitoids in batches of 5-20 individuals. We counted the number of females in each batch.

Parameter determination: We determined the fraction of females across all collected individuals.

Parasitoid lifespan experiment

Parameters determined: λ

Experimental procedure: We placed five female parasitoids in a patch with a radish plant. Every day, we recorded the number of dead parasitoids. We replicated this setup five times.

Parameter determination: We determined the lifespan as the mean age at death, weighted by the number of dead parasitoids found dead at that age.

Parasitoid dispersal experiment

Parameters determined: e_P, $N_{min,P}$

Experimental procedure: We connected two patches, each containing a single radish plant. We transferred 10 *L. erysimi* aphids into each patch. We added 2, 5 or 10 parasitoids into one of the patches (patch 1). We counted the number of parasitoids in both patches for three days. We replicated this experiment five times for each parasitoid density.

Parameter determination: We plotted the number of emigrated parasitoids per day (i.e., parasitoids in patch 2) against the number of parasitoids in patch 1 on the previous day (Figure S 5). We fitted a linear model, and obtained the minimum parasitoid population size for emigration, $N_{min,P}$, as the x-axis intercept as and the emigration rate, e_P as the slope.

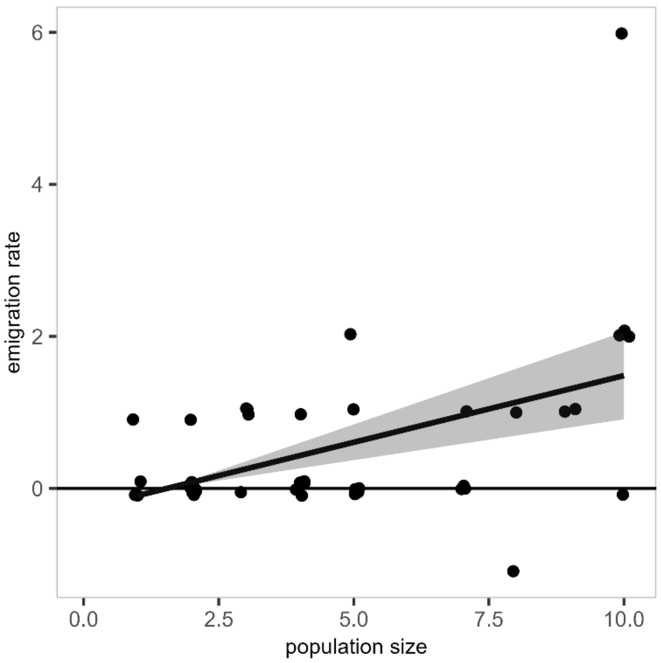

Figure S 5. Parasitoid dispersal experiment: emigration rate vs population size.

As we obtained all model parameters independently of each other, we refined their estimated ranges through iterative model simulations. First, we ran 1000 model simulations which reproduced either the single aphid or two aphid species response parameterisation experiments (see above). Every iteration, we sampled model parameters from uniform distributions defined by the confidence intervals obtained. We repeated this for communities of *B. brassicae* only, *L. erysimi* only and both aphid species together. Second, for every iteration, we calculated the error as the total absolute difference between the experimental observations and the corresponding model predictions. Third, we weighted each iteration by its error. We applied a linear weighting where iterations with the smallest and largest errors had weights of one and zero, respectively. Fourth, we selected 50% of iterations with the lower error. We did this independently for each community. Fifth, we obtained the interquartile range for each parameter weighted by the error in each iteration. In this final step, we considered all three communities collectively. This allowed us to determine the parameters that best reproduce both the single aphid and two aphid responses. We show model reproduction of the parameterisation experiments in Figure S 6 and the final model parameters determined form the experiments in Table S 1.

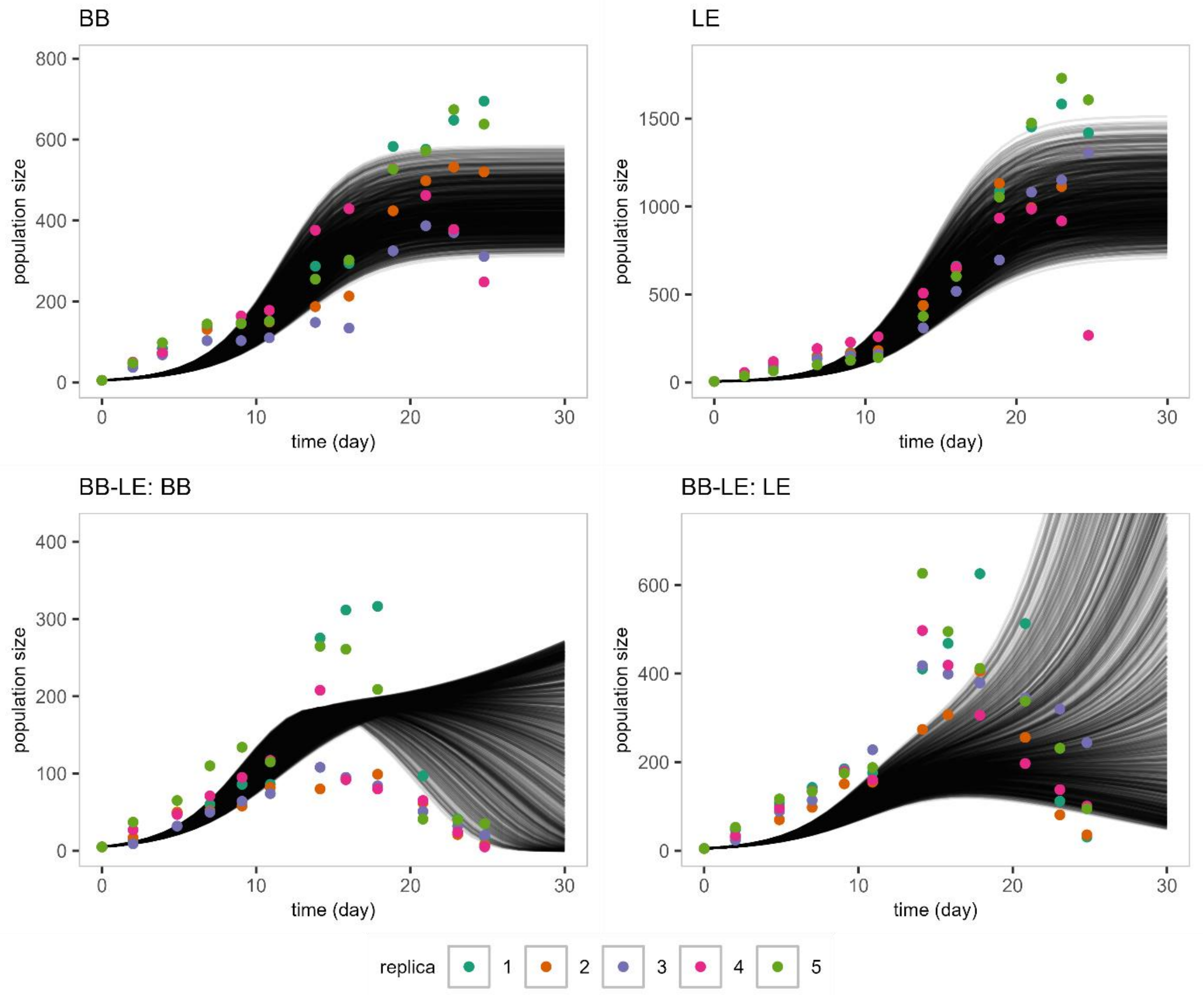

Figure S 6. Parameterisation experiments and their model simulations. Top panels show single aphid responses (left: *B. brassicae*, right: *L. erysimi*), whereas bottom panels depict the aphid responses in two-aphid communities. Points correspond to the experimental data, with colours indicating the replicas. Lines correspond to iterations of model simulations.

Table S 1. Model parameter values or ranges estimated from parameterisation experiments.

**model parameter**	**estimated value or range**		
	B. brassicae	***L. erysimi***	***D. rapae***
intrinsic growth rate, r_i	[0.314, 0.403]	[0.306, 0.397]	-
intraspecific competition, α_{ii}	[0.00070, 0.00104]	[0.00024, 0.00038]	-
interspecific competition, α_{ij}	[0.00092, 0.00115]	[0.00130, 0.00176]	-
parasitoid attack rate, β_i	[0.316, 0.717]	[0.208, 0.469]	-
maximum number of parasitised aphids, M_{max}	-	-	44
emigration rate, e_i [day^{-1}]	[0.018, 0.026]	[0.060, 0.101]	0.175
minimum population size for emigration, $N_{min,i}$	144	168	1.5
lifespan, λ [day]	-	-	7
time to emergence, τ [day]	-	-	16
fraction of females f	-	-	0.79

Non-experimental insect species

For the other species in our simulated food webs (i.e., aphid *Myzus persicae*, parasitoid wasp *Aphidius colemani* and hyperparasitoid wasp *Alloxysta fuscicornis*, Figure S 7), we estimated model parameters based on the values determined for *B. brassicae*, *L. erysimi* or *D. rapae*, previous studies and observations on this experimental system (Barbour et al., 2022), and expert opinion.

For *M. persicae*, we assumed the same intrinsic growth rate and intraspecific competition as *L. erysimi*, since the two species reach similar carrying capacities when reared separately. We assumed the interspecific competition by *M. persicae* on the other two aphids and vice versa to be the average of interspecific competition of *B. brassicae* and *L. erysimi* (i.e. $\alpha_{3j} = \alpha_{i3} = (\alpha_{12} + \alpha_{21})/2$, where subscripts 1,2 and 3 refer to *B. brassicae*, *L. erysimi* and *M. persicae*, respectively). This resulted in *M. persicae* having the highest competitive ability our of the three aphid species (quantified using the approach proposed by Hart et al., 2018), and *B. brassicae* is the lowest, which is consistence with experimental observations. For the dispersal parameters of *M. persicae* we adopted the average of the corresponding values of the other two aphids.

D. rapae has the strongest preference for parasitising *B. brassicae*, and the weakest for *M. persicae*. Therefore, we assumed $\beta_{31} = 2/3\,\beta_{21}$ (where the first subscript – 2, 3 – refers to the aphid species – *L. erysimi*, *M. persicae*, whereas the second – 1 – refers to the parasitoid *D. rapae*). *A. colemani* has a strong preference for *M. persicae*, but has been observed to parasitise *B. brassicae* and *L. erysimi* as well. Therefore we assumed $\beta_{12} = \beta_{22} = 1/3\,\beta_{21}$ and $\beta_{32} = \beta_{11}$ (where the first subscript – 1, 2, 3 – refers to the aphid species – *B. brassicae* , *L. erysimi*, *M. persicae*, whereas the second – 2 – refers to the parasitoid *A. colemani*).

The hyperparasitoid *A. fuscicornis* has a preference for parasitising *D. rapae* over *A. colemani*. We assumed the hyperparasitoid attack rates, γ_i, to be the same as the parasitoid-aphid values such that the attack rate on *D. rapae* and *A. colemani* is $\gamma_1 = \beta_{11}$ and $\gamma_2 = \beta_{21}$, respectively. All other model parameters were assumed to be the same for the two parasitoid and hyperparasitoid species.

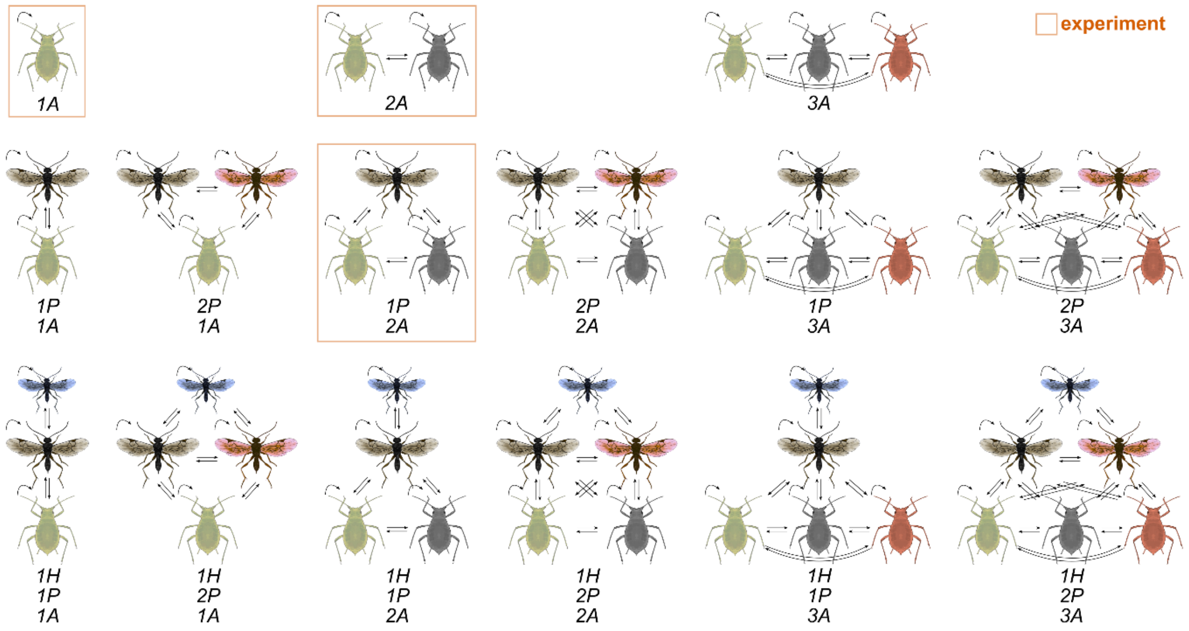

Figure S 7. 15 simulated communities increasing in food-web complexity from a single aphid species to three aphid, two parasitoid wasp and one hyperparasitoid wasp species. The experimental communities are indicated by the orange box. The communities are named according to the number of aphid (A), parasitoid (P) and hyperparasitoid (H) species. The aphid icons correspond to *Brevicoryne brassicae* (green), *Lipaphis erysimi* (grey), and *Myzus persicae* (red). The parasitoid wasp icons represent *Diaeretiella rapae* (grey) and *Aphidius colemani* (red). The hyperparasitoid wasp is *Alloxysta fuscicornis*. The sizes and colours of insect icons were chosen for illustrative purposes.

Experimental landscapes and communities

Experimental results

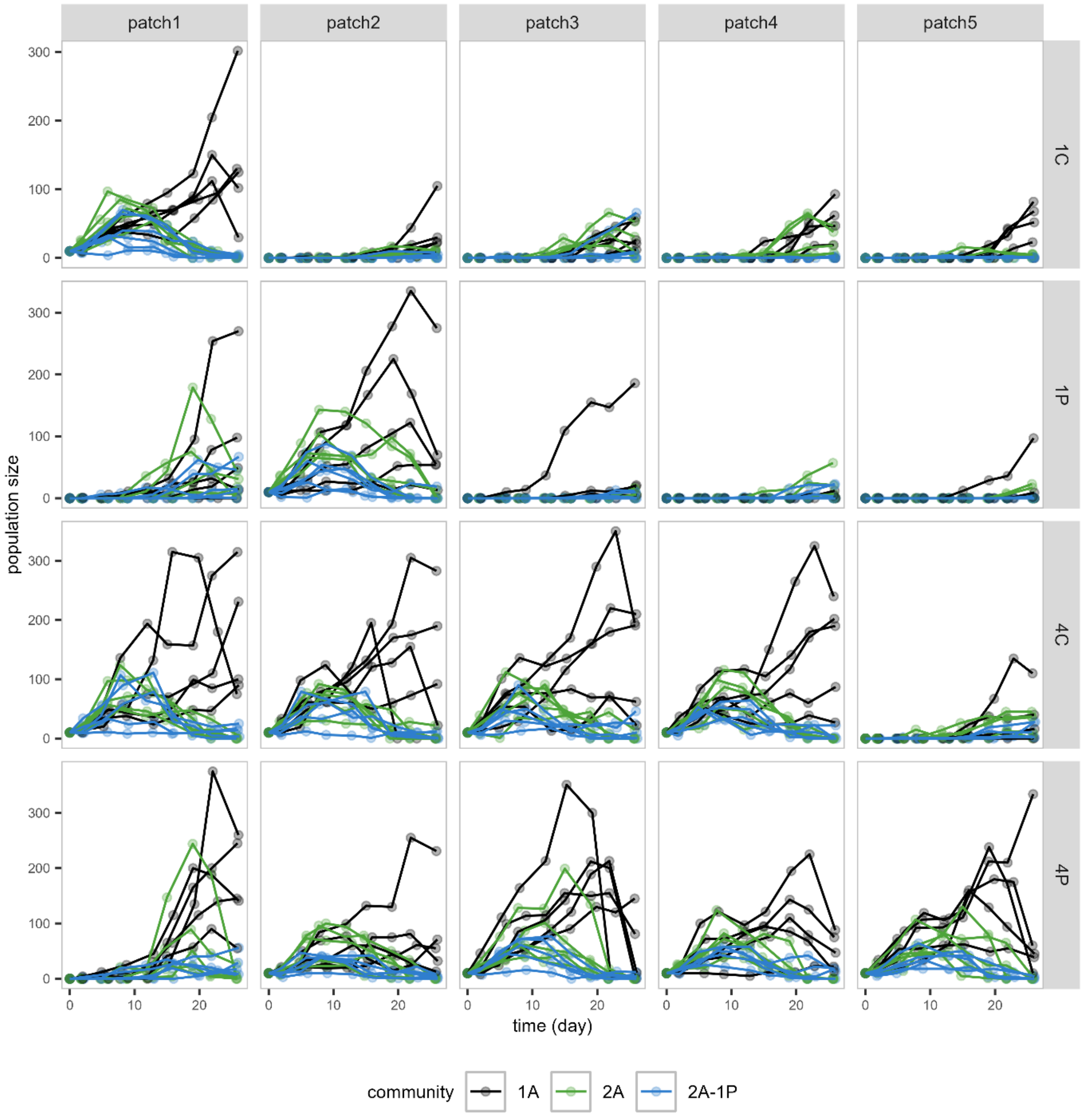

Figure S 8. Observed population size of aphid *B. brassicae* with time. Lines join observations belonging to the same experimental replica. Colours indicate insect communities. Panels correspond to different landscapes (rows) and patches (columns). Patch1 is the central patch.

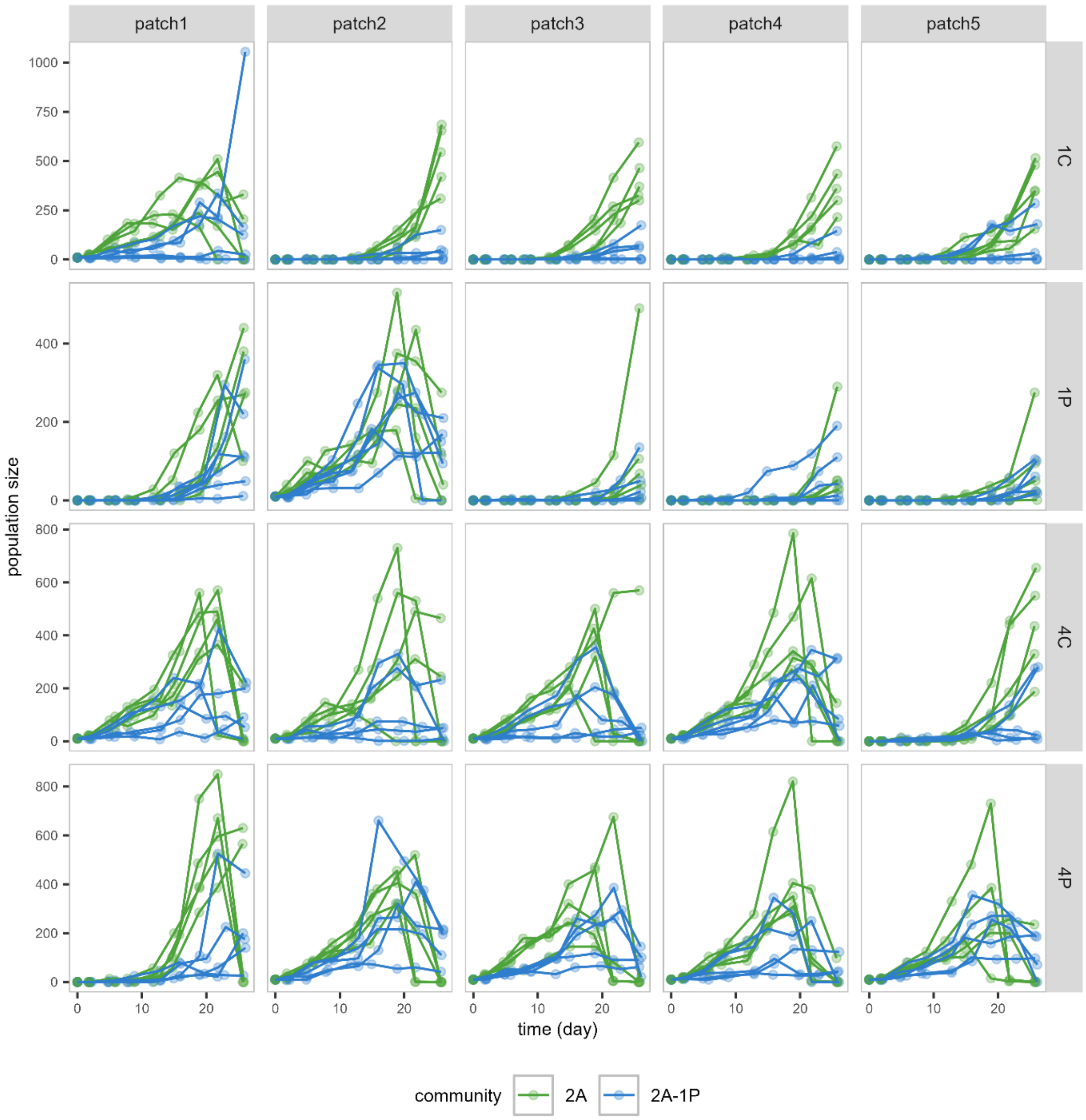

Figure S 9. Observed population size of aphid *L. erysimi* with time. Lines join observations belonging to the same experimental replica. Colours indicate insect communities. Panels correspond to different landscapes (rows) and patches (columns). Patch1 is the central patch.

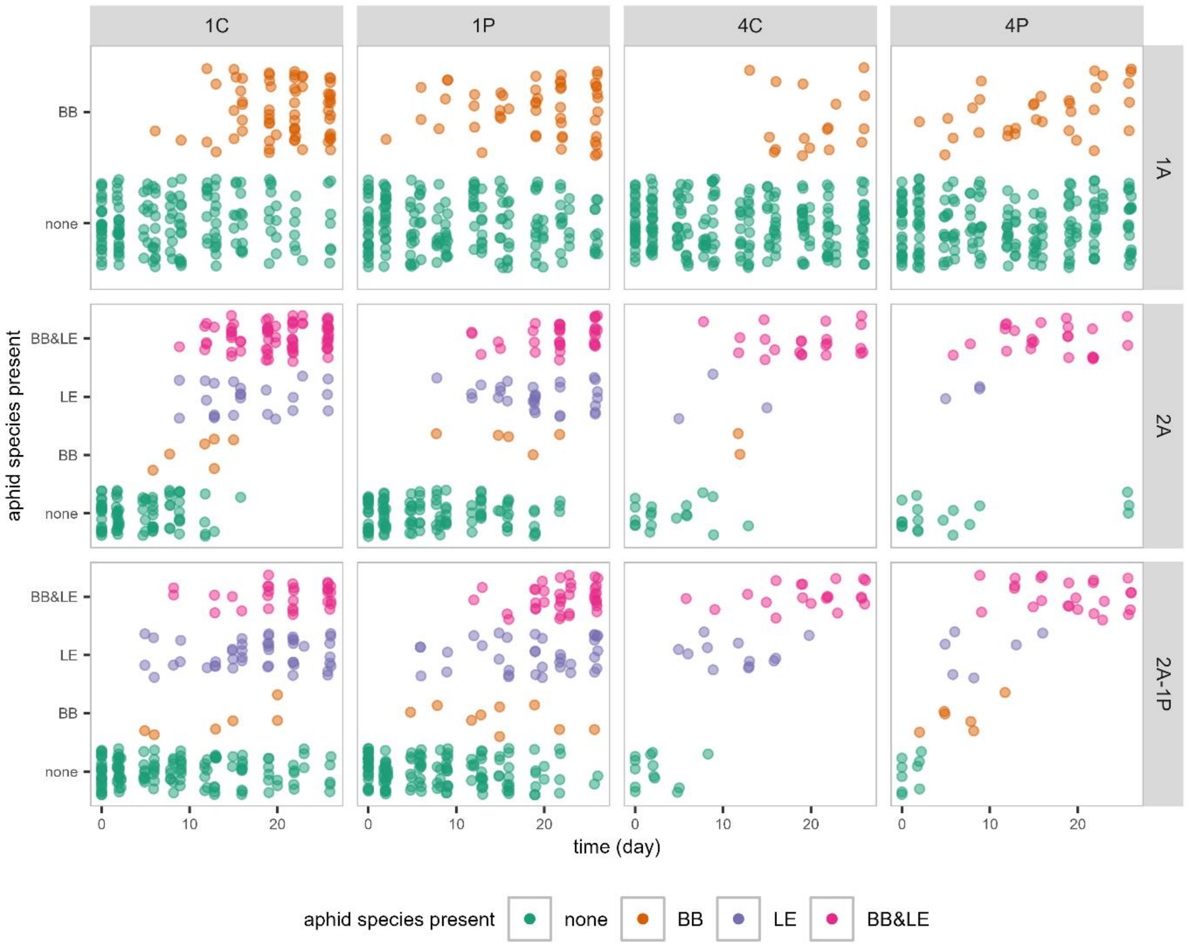

Figure S 10. Observed aphid species presence in initially *empty* patches with time (BB - *B. brassicae*, LE - *L. erysimi*). Panels correspond to different landscapes (columns) and communities (rows). Each point corresponds to an initially empty patch in a replicate experiment.

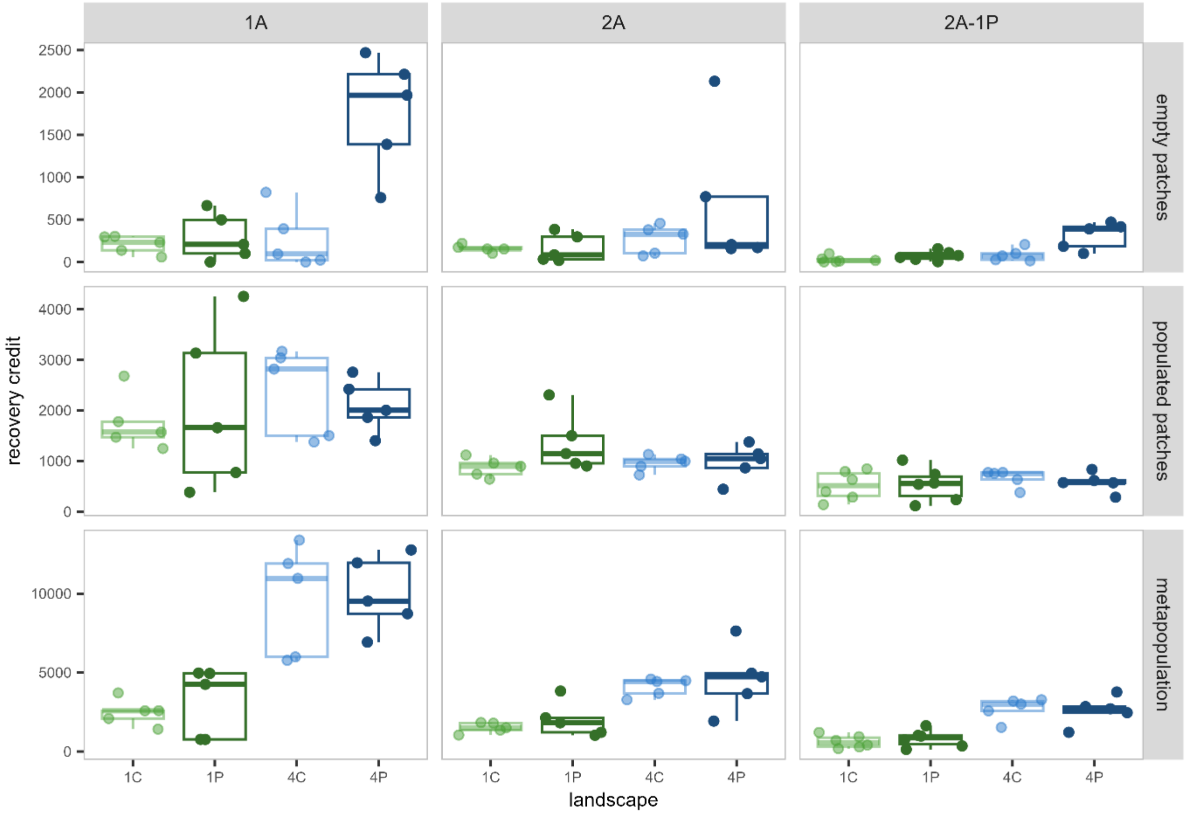

Figure S 11. Experimental recovery of aphid *B. brassicae*. Points plot the recovery credit calculated from experimental data. Panels correspond to different scales (rows) and communities (columns).

Table S 2. ANOVA of the effects for number of communities, location of communities, and community food web on the recovery credit of aphid *B. brassicae* in initially *empty* patches (experimental results).

	Degrees of freedom	Sum of Squares	Mean Square	F-value	P-value
number of communities	1	20.752	20.7522	10.7836	0.001874
location of communities	1	12.926	12.9255	6.7166	0.012489
community food web	2	20.96	10.4801	5.4458	0.00725
number of communities x location of communities	1	11.949	11.9489	6.2091	0.016071
location of communities x community food web	2	1.473	0.7364	0.3826	0.684035
location of communities x community food web	2	6.034	3.0168	1.5676	0.218613
number of communities x location of communities x community food web	2	9.038	4.5189	2.3482	0.105994
Residuals	50	96.221	1.9244		

Table S 3. ANOVA of the effects for number of communities, location of communities, and community food web on the recovery credit of aphid *B. brassicae* in initially *populated* patches (experimental results).

	Degrees of freedom	Sum of Squares	Mean Square	F-value	P-value
number of communities	1	0.6986	0.6986	2.5957	0.1135
location of communities	1	0.001	0.001	0.0038	0.9513
community food web	2	17.5011	8.7506	32.5134	9.00e-10
number of communities x location of communities	1	0.1189	0.1189	0.442	0.5092
location of communities x community food web	2	0.6193	0.3097	1.1506	0.3247
location of communities x community food web	2	0.2785	0.1393	0.5174	0.5992
number of communities x location of communities x community food web	2	0.1522	0.0761	0.2827	0.7549
Residuals	50	13.4568	0.2691		

Table S 4. ANOVA of the effects for number of communities, location of communities, and community food web on the recovery credit of aphid *B. brassicae metapopulation* (experimental results).

	Degrees of freedom	Sum of Squares	Mean Square	F-value	P-value
number of communities	1	27.2292	27.2292	87.7044	1.39E-12
location of communities	1	0.0751	0.0751	0.242	0.6249
community food web	2	19.9125	9.9562	32.0687	1.09E-09
number of communities x location of communities	1	0.0507	0.0507	0.1634	0.6878
location of communities x community food web	2	0.9552	0.4776	1.5383	0.2247
location of communities x community food web	2	0.0209	0.0104	0.0336	0.967
number of communities x location of communities x community food web	2	0.0992	0.0496	0.1597	0.8528
Residuals	50	15.5233	0.3105		

Model simulations

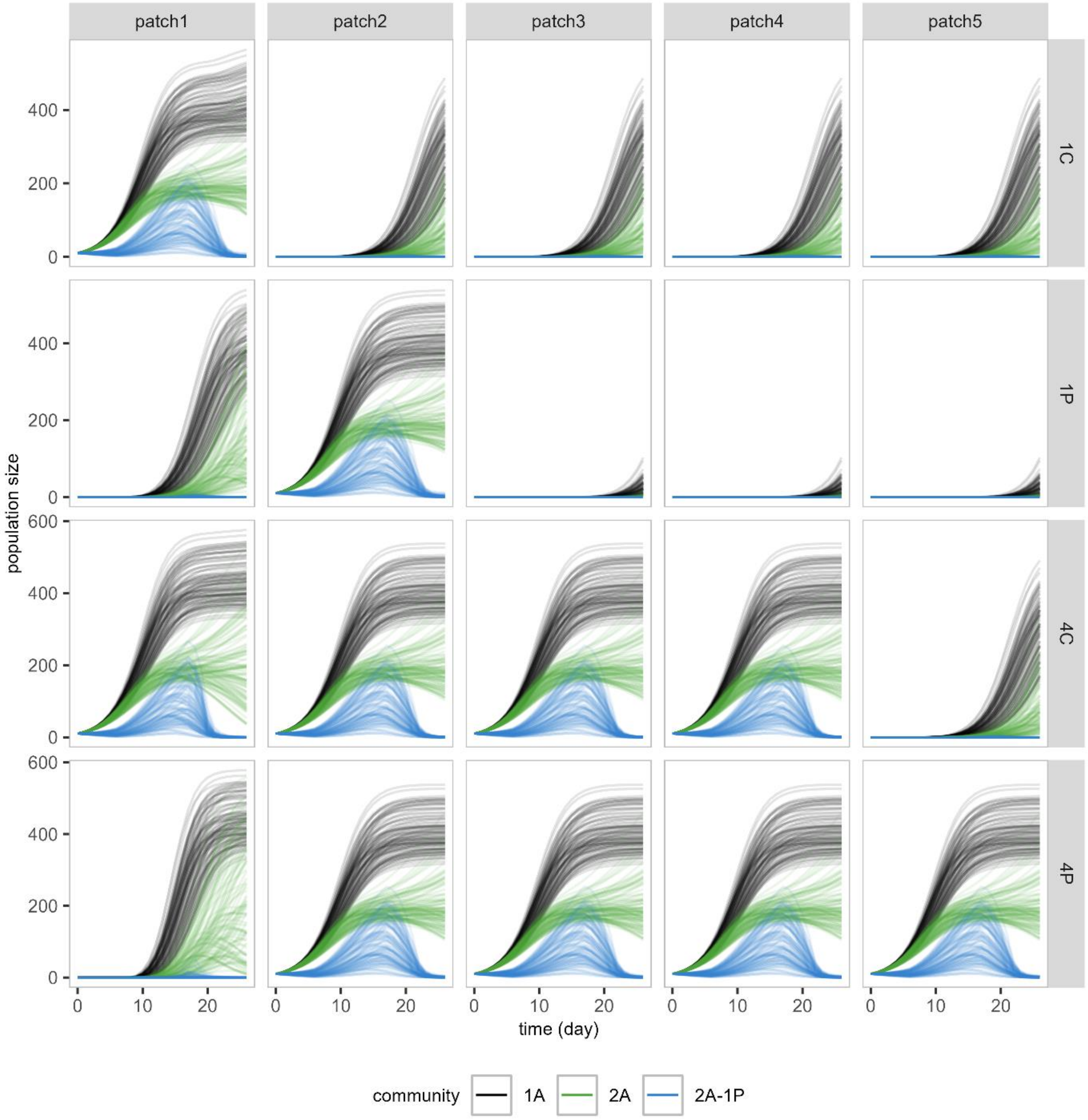

Figure S 12. Simulated population size of aphid *B. brassicae* with time. Lines join observations belonging to the same simulation replica. Colours indicate insect communities. Panels correspond to different landscapes (rows) and patches (columns). Patch1 is the central patch.

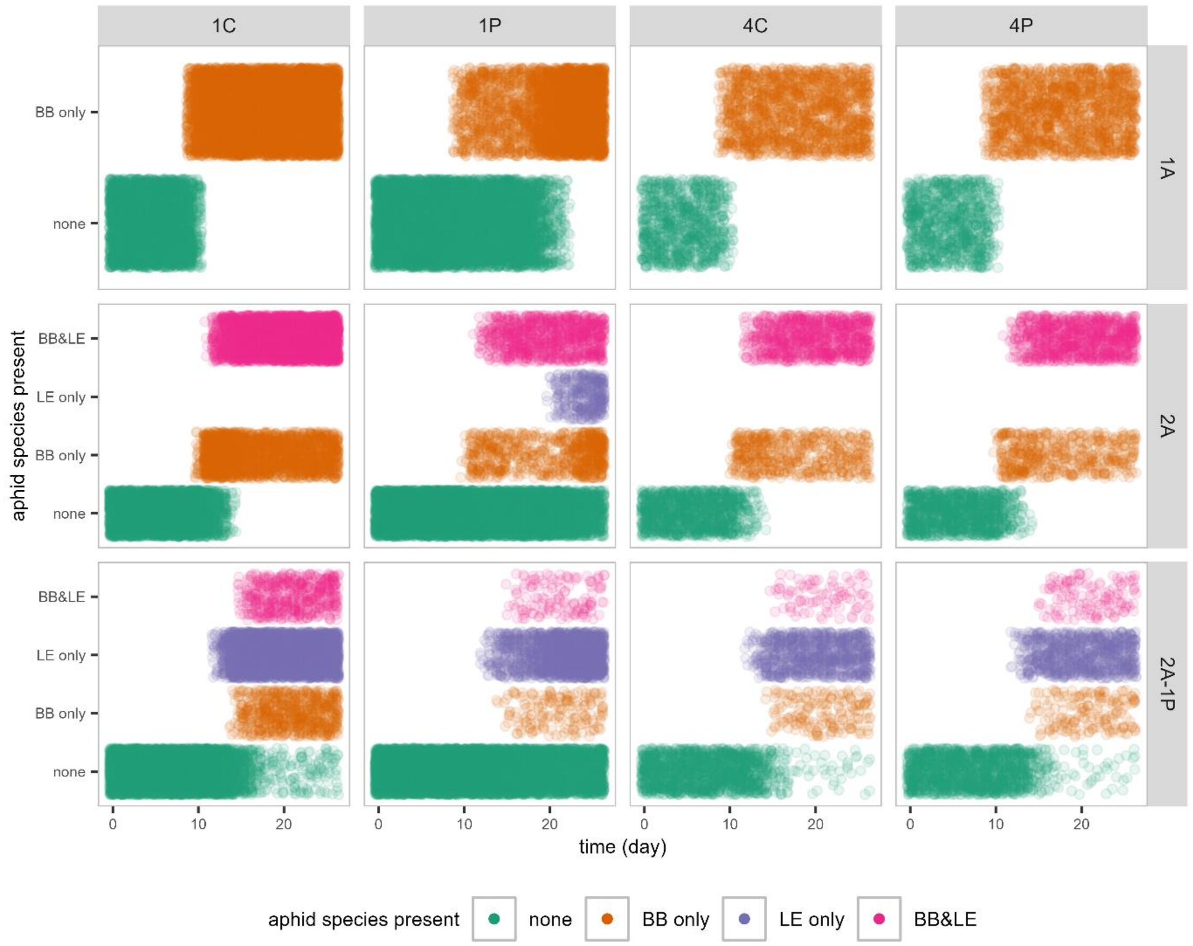

Figure S 13. Simulated aphid species presence in initially *empty* patches with time (BB - *B. brassicae*, LE - *L. erysimi*). Panels correspond to different landscapes (columns) and communities (rows). Each point corresponds to an initially empty patch in a replicate experiment.

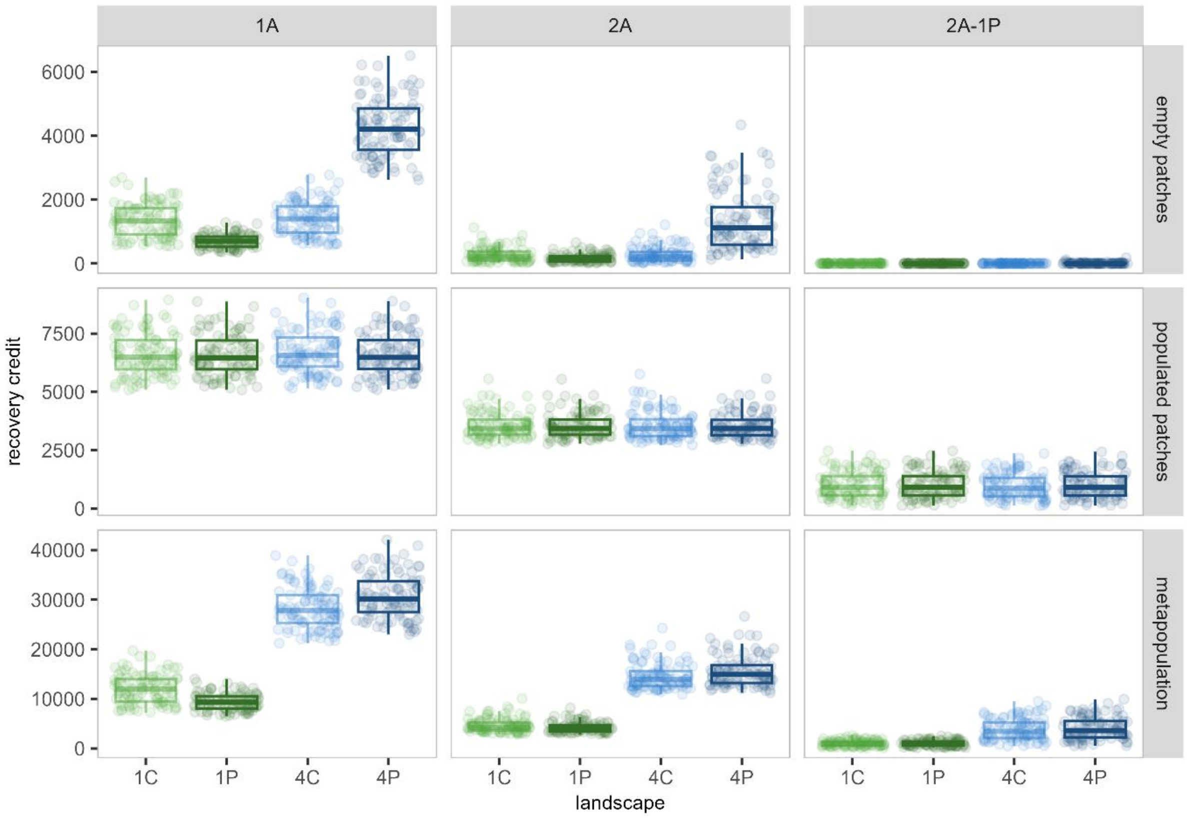

Figure S 14. Simulated recovery of aphid *B. brassicae*. Points plot the recovery credit calculated from simulated data. Panels correspond to different scales (rows) and communities (columns).

Table S 5. ANOVA of the effects for number of communities, location of communities, and community food web on the recovery credit of aphid *B. brassicae* in initially *empty* patches (simulation results).

	Degrees of freedom	Sum of Squares	Mean Square	F-value	P-value
number of communities	1	157.9	157.9	270.021	<2.2e-16
location of communities	1	35.7	35.7	61.128	1.173e-14
community food web	2	10131.6	5065.8	8662.204	<2.2e-16
number of communities x location of communities	1	157.6	157.6	269.482	<2.2e-16
location of communities x community food web	2	44.9	22.5	38.409	<2.2e-16
location of communities x community food web	2	15.9	8.0	13.599	1.447e-06
number of communities x location of communities x community food web	2	34.8	17.4	29.733	2.511e-13
Residuals	1188	694.8	0.6		

Table S 6. ANOVA of the effects for number of communities, location of communities, and community food web on the recovery credit of aphid *B. brassicae* in initially *populated* patches (simulation results).

	Degrees of freedom	Sum of Squares	Mean Square	F-value	P-value
number of communities	1	0.03	0.03	0.1604	0.6889
location of communities	1	0.01	0.01	0.0558	0.8133
community food web	2	888.59	444.29	2820.7153	<2e-16
number of communities x location of communities	1	0.01	0.01	0.07	0.7914
location of communities x community food web	2	0.11	0.05	0.3414	0.7108
location of communities x community food web	2	0.07	0.03	0.2103	0.8104
number of communities x location of communities x community food web	2	0.06	0.03	0.1953	0.8226
Residuals	1188	187.12	0.16		

Table S 7. ANOVA of the effects for number of communities, location of communities, and community food web on the recovery credit of aphid *B. brassicae metapopulation* (simulation results).

	Degrees of freedom	Sum of Squares	Mean Square	F-value	P-value
number of communities	1	435.66	435.66	2527.5007	< 2.2e-16
location of communities	1	0.1	0.1	0.5774	0.447490
community food web	2	1131.15	565.57	3281.1747	< 2.2e-16
number of communities x location of communities	1	2.17	2.17	12.5964	0.000402
location of communities x community food web	2	5.15	2.58	14.9515	3.86E-07
location of communities x community food web	2	0.52	0.26	1.5076	0.221874
number of communities x location of communities x community food web	2	0.87	0.43	2.5191	0.080961
Residuals	1188	204.77	0.17		

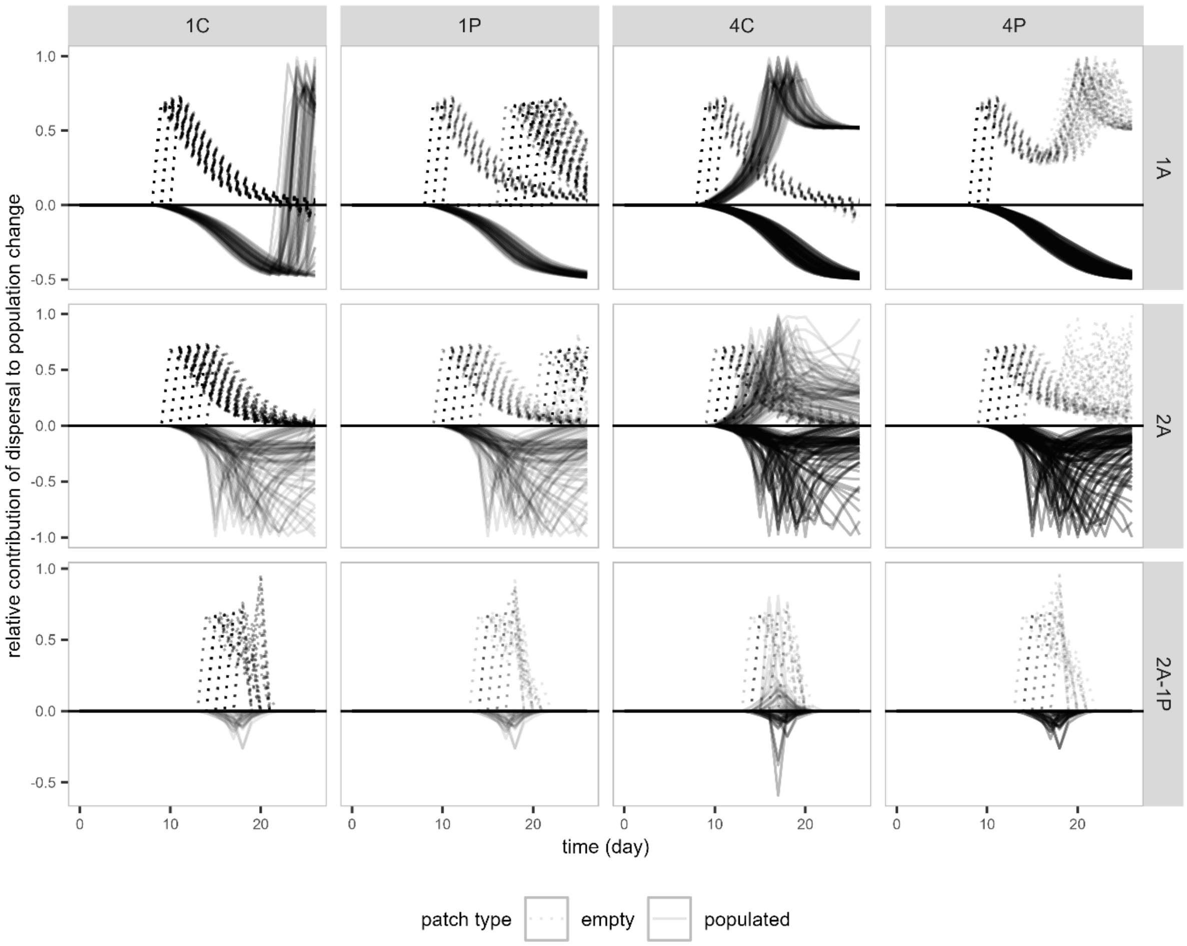

Figure S 15. Relative contribution of dispersal to population change of aphid *B. brassicae* with time. Positive and negative contributions indicate net immigration and emigration, respectively. Panels correspond to insect communities (rows) in different landscapes (columns). Lines join observations belonging to the same simulation replica. Dotted and solid lines depict initially *empty* and *populated* patches, respectively.

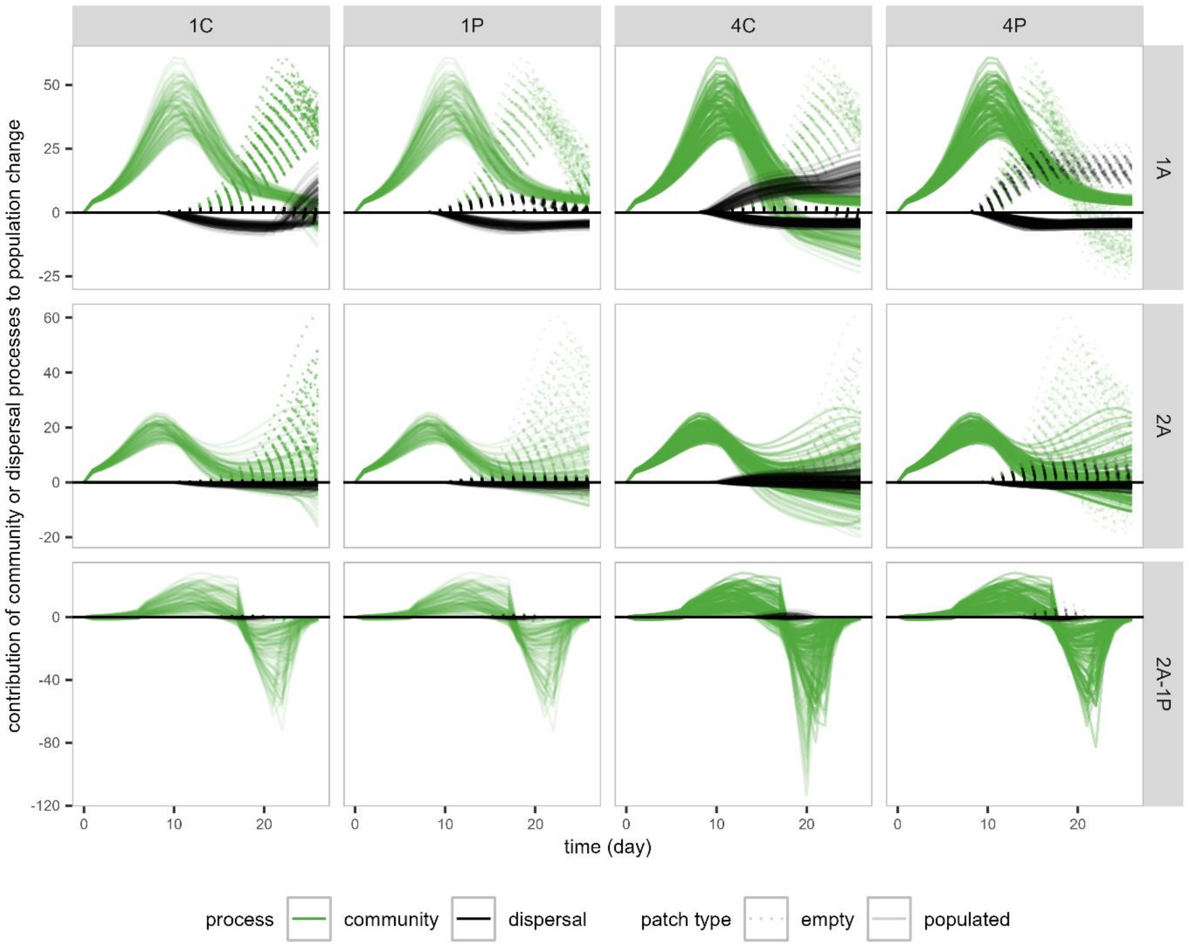

Figure S 16. Contributions of community and dispersal processes to population change of aphid *B. brassicae* with time. Panels correspond to insect communities (rows) in different landscapes (columns). Lines join observations belonging to the same simulation replica. Line colours indicate community (green) or dispersal (black) contributions. Dotted and solid lines depict initially *empty* and *populated* patches, respectively.

Larger landscapes and communities

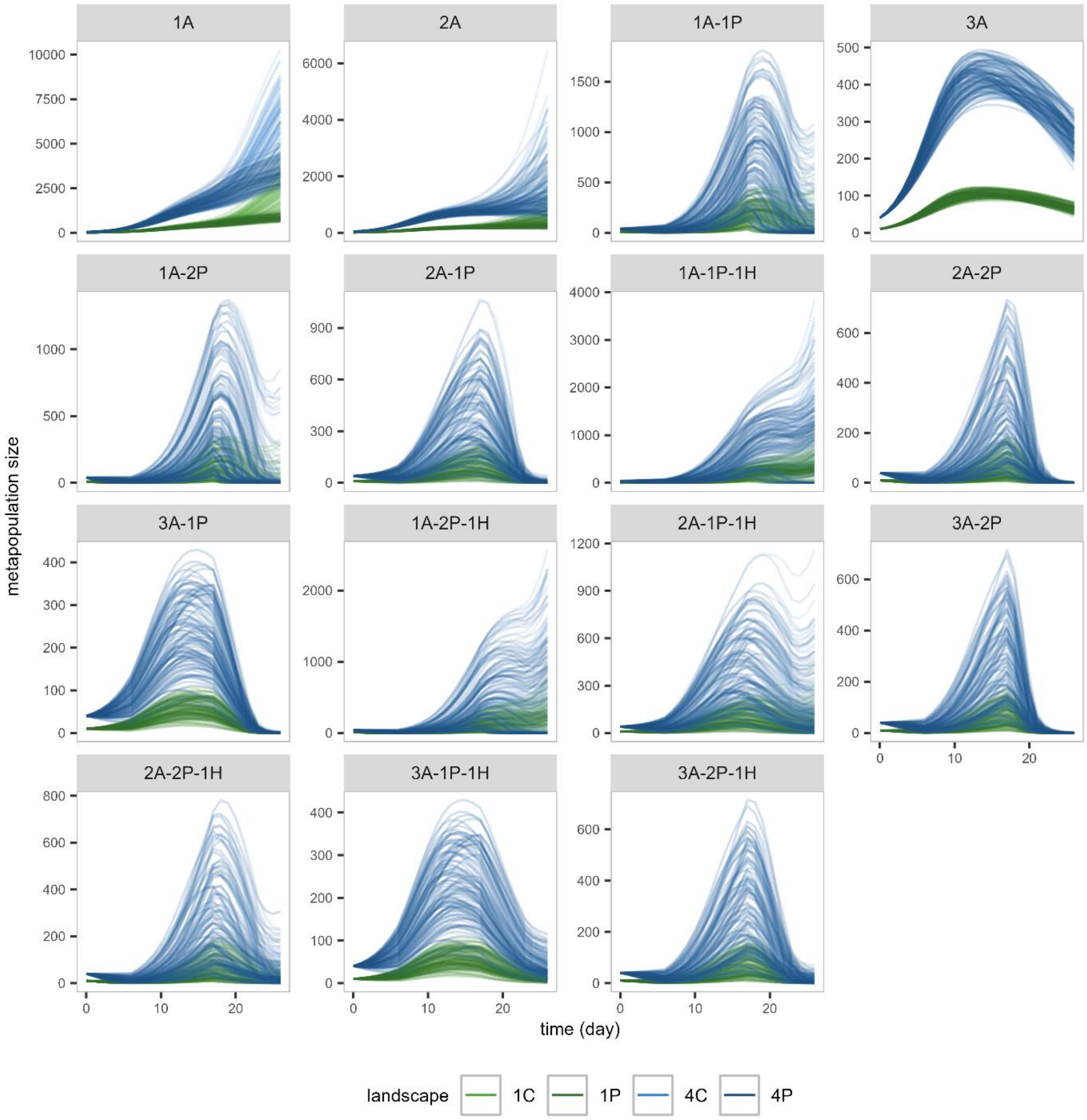

Figure S 17. Simulated *metapopulation* size of aphid *B. brassicae* with time. Lines join observations belonging to the same simulation replica on larger landscapes and communities. Colours indicate landscapes. Panels correspond to different communities.

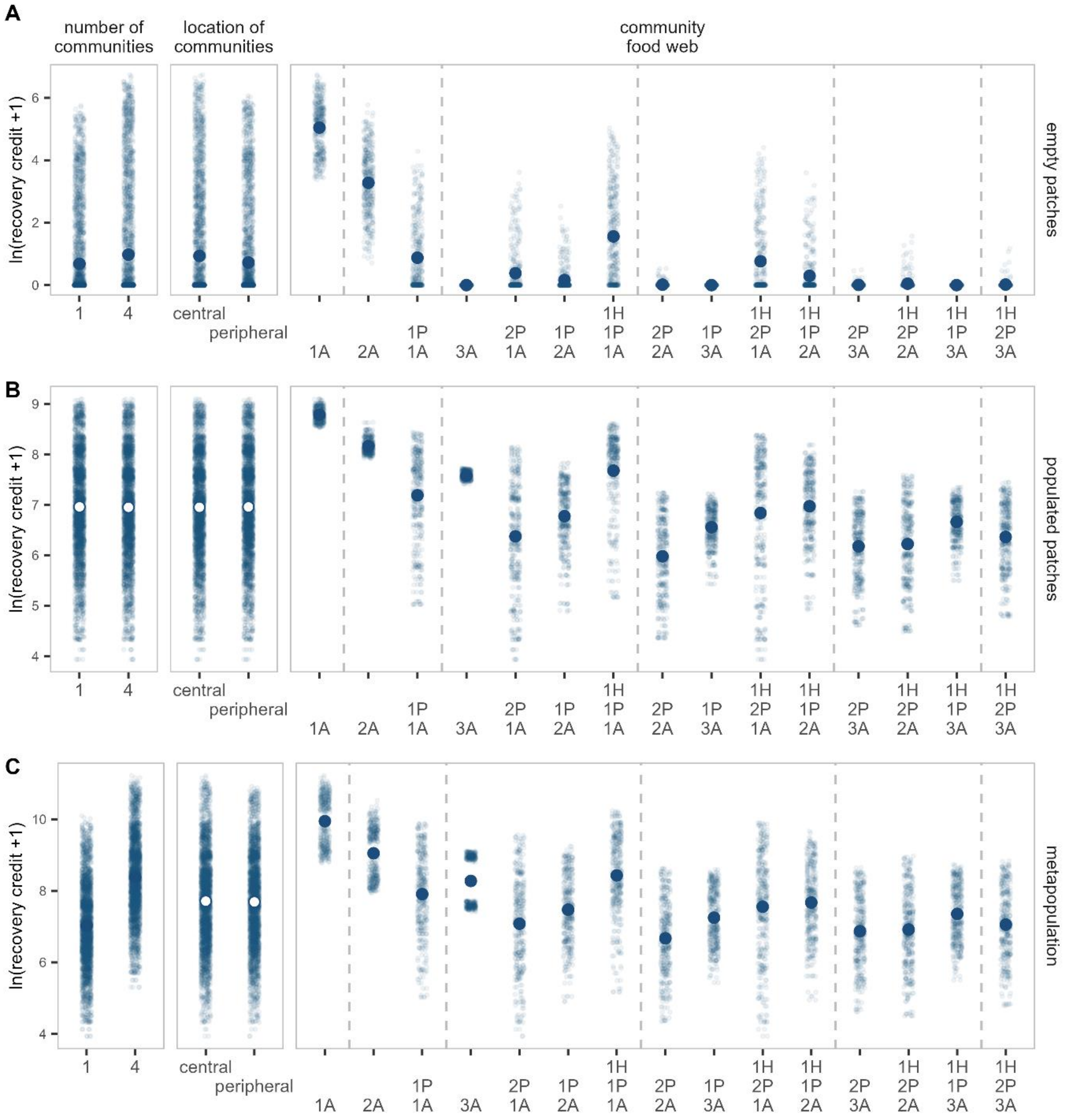

Figure S 18. Recovery of aphid *B. brassicae* (A) in initially *empty* patches, (B) in initially *populated* patches, and (C) *metapopulation*. Panels show the effects of initial number of communities (left), location of initial communities (middle), and community food web (right) on the recovery credit ($ln(x + 1)$ transformed). Smaller points represent recovery credit calculated from model simulations on larger landscapes and communities. Larger points and vertical lines depict average linear model predictions and their 95% confidence intervals, respectively. Full and empty points indicate statistically significant and nonsignificant effects, respectively.

Table S 8. ANOVA of the effects for number of communities, location of communities, and community food web on the recovery credit of aphid *B. brassicae* in initially *empty* patches (simulated results on larger landscapes and communities).

	Degrees of freedom	Sum of Squares	Mean Square	F-value	P-value
number of communities	1	127.6	127.62	304.0566	< 2e-16
location of communities	1	66.5	66.49	158.4077	< 2e-16
community food web	14	11967.1	854.79	2036.539	< 2e-16
number of communities x location of communities	1	1.6	1.56	3.7247	0.05366
location of communities x community food web	14	252.3	18.02	42.9445	< 2e-16
location of communities x community food web	14	79.9	5.71	13.6026	< 2e-16
number of communities x location of communities x community food web	14	5.1	0.37	0.8704	0.59147
Residuals	5940	2493.2	0.42		

Table S 9. ANOVA of the effects for number of communities, location of communities, and community food web on the recovery credit of aphid *B. brassicae* in initially *populated* patches (simulated results on larger landscapes and communities).

	Degrees of freedom	Sum of Squares	Mean Square	F-value	P-value
number of communities	1	0.1	0.136	0.2677	0.6049
location of communities	1	0.1	0.076	0.1499	0.6986
community food web	14	3540.5	252.896	499.0337	<2e-16
number of communities x location of communities	1	0.1	0.142	0.2794	0.5971
location of communities x community food web	14	0.1	0.005	0.0096	1
location of communities x community food web	14	0	0.002	0.0046	1
number of communities x location of communities x community food web	14	0.1	0.005	0.0103	1
Residuals	5940	3010.2	0.507		

Table S 10. ANOVA of the effects for number of communities, location of communities, and community food web on the recovery credit of aphid *B. brassicae metapopulation* (simulated results on larger landscapes and communities).

	Degrees of freedom	Sum of Squares	Mean Square	F-value	P-value
number of communities	1	2791.8	2791.76	5268.898	<2e-16
location of communities	1	0.8	0.75	1.4178	0.2338
community food web	14	4531.3	323.66	610.8501	<2e-16
number of communities x location of communities	1	0.7	0.67	1.2561	0.2624
location of communities x community food web	14	0.6	0.04	0.0785	1
location of communities x community food web	14	6.4	0.45	0.8563	0.6073
number of communities x location of communities x community food web	14	0.6	0.04	0.0829	1
Residuals	5940	3147.3	0.53		

References

Agrawal, A. A. 2004. Plant defense and density dependence in the population growth of herbivores. *The American Naturalist,* 164**,** 113-120.

Barbour, M. A., Kliebenstein, D. J. & Bascompte, J. 2022. A keystone gene underlies the persistence of an experimental food web. *Science (New York, N.Y.),* 376**,** 70–73.

Hart, S. P., Freckleton, R. P. & Levine, J. M. 2018. How to quantify competitive ability. *Journal of Ecology,* 106**,** 1902–1909.

Hodgson, C. 1991. Dispersal of apterous aphids (homoptera: Aphididae) from their host plant and its significance. *Bulletin of Entomological Research,* 81**,** 417-427.

Rosenbaum, B., Li, J., Hirt, M. R., Ryser, R. & Brose, U. 2024. Towards understanding interactions in a complex world: Design and analysis of multi-species functional response experiments. *Methods in Ecology and Evolution,* 15**,** 1704-1719.

Soni, S. & Kumar, S. 2021. Efficacy of the parasitoid, diaeretiella rapae (mcintosh) (hymenoptera: Braconidae) against myzus persicae (sulzer) (hemiptera: Aphididae) infesting rapeseed-mustard. *Journal of Asia-Pacific Entomology,* 24**,** 912–917.
